# Inhibition of p38 MAPK or immunoproteasome overcomes resistance of chronic lymphocytic leukemia cells to Bcl-2 antagonist venetoclax

**DOI:** 10.1038/s41419-022-05287-6

**Published:** 2022-10-08

**Authors:** Damjan Avsec, Marja Škrlj Miklavčič, Tilen Burnik, Maša Kandušer, Maruša Bizjak, Helena Podgornik, Irena Mlinarič-Raščan

**Affiliations:** 1 University of Ljubljana, Faculty of Pharmacy, SI-1000 Ljubljana, Slovenia; 2 University Medical Centre Ljubljana, Department of Haematology, SI-1000 Ljubljana, Slovenia

**Keywords:** Chronic lymphocytic leukaemia, Chronic lymphocytic leukaemia

## Abstract

Chronic lymphocytic leukemia (CLL) is a hematological neoplasm of CD19-positive mature-appearing B lymphocytes. Despite the clinical success of targeted therapies in CLL, the development of resistance diminishes their therapeutic activity. This is also true for the Bcl-2 antagonist venetoclax. We investigated the molecular mechanisms that drive venetoclax resistance in CLL, with a clear focus to provide new strategies to successfully combat it. Activation of CLL cells with IFNγ, PMA/ionomycin, and sCD40L diminished the cytotoxicity of venetoclax. We demonstrated that the metabolic activity of cells treated with 1 nM venetoclax alone was 48% of untreated cells, and was higher for cells co-treated with IFNγ (110%), PMA/ionomycin (78%), and sCD40L (62%). As of molecular mechanism, we showed that PMA/ionomycin and sCD40L triggered translocation of NFκB in primary CLL cells, while IFNγ activated p38 MAPK, suppressed spontaneous and venetoclax-induced apoptosis and induced formation of the immunoproteasome. Inhibition of immunoproteasome with ONX-0914 suppressed activity of immunoproteasome and synergized with venetoclax against primary CLL cells. On the other hand, inhibition of p38 MAPK abolished cytoprotective effects of IFNγ. We demonstrated that venetoclax-resistant (MEC-1 VER) cells overexpressed p38 MAPK and p-Bcl-2 (Ser70), and underexpressed Mcl-1, Bax, and Bak. Inhibition of p38 MAPK or immunoproteasome triggered apoptosis in CLL cells and overcame the resistance to venetoclax of MEC-1 VER cells and venetoclax-insensitive primary CLL cells. In conclusion, the p38 MAPK pathway and immunoproteasome represent novel targets to combat venetoclax resistance in CLL.

## Introduction

Chronic lymphocytic leukemia (CLL) is a hematological neoplasm that is characterized by the expansion of CD5-, CD19-positive mature-appearing monoclonal B lymphocytes in the blood, bone marrow, and lymph nodes, which are resistant to apoptosis [1, 2]. Therefore, therapies that promote apoptosis of CLL cells successfully made the transition from bench to bedside. One such therapy is the antagonist of the anti-apoptotic protein Bcl-2, venetoclax [3, 4]. However, along with the clinical success of venetoclax, its prolonged use has also resulted in the development of resistance [4, 5], which defines the need to understand such resistance-driving mechanisms in CLL.

The maintenance of tumor cells strongly depends on the presence of a supportive tumor microenvironment. This is especially true in CLL, where nurse-like cells, stromal cells, NK cells, and T cells release chemokines and cytokines, that support the survival and proliferation of clones [6]. Tumor microenvironment nurtures and protects CLL cells from cytotoxic stimuli, as well as supports the growth and expansion of clones [6–8], and has thus been recognized as an important aspect of drug resistance.

Interferon-gamma (IFNγ) is one of the prosurvival factors in the tumor microenvironment of CLL cells [9], and it is of particular interest for the following reasons. The serum concentrations of IFNγ are higher in patients with CLL compared to healthy controls [10, 11], which might be due, at least in part, to the production of IFNγ by CLL cells [11]. IFNγ protects CLL cells from spontaneous apoptosis and promotes CLL cell survival [11–14], and it can also induce proliferation and differentiation of CLL cells [15]. More importantly, IFNγ can rescue CLL cells from apoptosis triggered by targeted therapies, such as ibrutinib [16], and it is likely implicated in resistance to venetoclax through an Mcl-1–dependent mechanism [14, 17, 18]. IFNγ is a type II IFN and it binds to the IFNγ receptor (IFNγR), a heterodimer of IFNγRI and IFNγRII. Following the binding of IFNγ to IFNγR, the signals are transduced mainly, although not exclusively, through the Janus kinase (JAK)/signal transducer and activator of transcription (STAT) pathways [19]. Following the treatment of cells with IFNγ, some studies have also reported activation of mitogen-activated protein kinases (MAPKs) (i.e., MEK/ERK1/2, JNK, p38 MAPK), phosphatidylinositol 3-kinase, calcium/calmodulin-dependent kinase II, and the nuclear factor kappa B (NFκB) pathway [20].

IFNγ can induce the formation of the immunoproteasome, which is the predominant form of the proteasome found in cells of the immune system, including B lymphocytes. In the presence of proinflammatory cytokines (e.g. IFNγ, TNFα) constitutive proteasome subunits β1, β2, and β5 are replaced by β1i (LMP2), β2i (MECL1), and β5i (LMP7), respectively, to form the immunoproteasome [21, 22]. The constitutive proteasome modulates the activity of the canonical NFκB pathway through the degradation of the inhibitor of κB [23–25], and it has been suggested that this is also the case for the immunoproteasome [26]. Activation of B-cell receptor (BCR) and NFκB promotes cell survival in the tumor microenvironment of CLL, and thus constitutes an important mechanism of drug resistance [27]. We previously demonstrated that perturbation of the NFκB pathway can enhance the cytotoxicity of targeted therapies against CLL cells, including that of the Bcl-2 antagonist venetoclax [28]. What is more, the NFκB pathway is at the center of BCR- [18] and CD40L-mediated resistance of CLL cells to venetoclax [29, 30]. CD40L can also trigger alternative NFκB signaling to mediate resistance to BCR inhibitors [31]. It has been suggested that the p38 MAPK pathway is required for CD40-induced NFκB activation in B lymphocytes [32], thereby linking p38 MAPK to the NFκB pathway and venetoclax resistance.

However, a role for p38 MAPK in CLL is controversial. It has been shown that CD40 ligation or stimulation of BCR regulates the proliferation of B cells through p38 MAPK [32–34]. Furthermore, p38 MAPK is constitutively activated in CLL cells [35], which was shown to be essential for the survival of primary CLL cells cultured on bone-marrow-derived stromal cells, indicating that activated p38 MAPK promotes prosurvival signals [35] and is also likely to foster chemoresistance in B-cell non-Hodgkin lymphoma [36]. However, several studies have contradicted such a cytoprotective role of p38 MAPK in CLL. Huelsemann et al. showed that inhibition of p38 MAPK in CLL cells attenuated not only spontaneous apoptosis but also BH3-mimetic-induced apoptosis [37]. Similarly, the promotion of apoptosis in CLL cells through p38 MAPK was also shown for the anti-CD20 antibody rituximab [38] and for cyclin-dependent kinase inhibitor [39], as their cytotoxicities were significantly reduced by a p38 MAPK inhibitor. Together, these findings underline the need to further address the role of p38 MAPK in the maintenance and resistance of CLL cells.

In the present study, we investigated how different activators of CLL cells affect the cytotoxicity of venetoclax, with particular focus on cytoprotective and anti-apoptotic properties of IFNγ. Here, we shed light on the importance of the immunoproteasome and p38 MAPK in IFNγ-mediated and drug-induced resistance of CLL cells to venetoclax, and the potential of targeting the immunoproteasome and p38 MAPK to successfully overcome this resistance.

## Materials and methods

### Compounds and cytokines

Venetoclax (Cat. N° HY-15531), BIRB796 (doramapimod; HY-10320), SB203580 (HY-10256), M3258 (HY-111790), ML604440 (HY-114170), marizomib (HY-10985), dithiothreitol (HY-15917; DTT), ONX-0914 (HY-13207), quinoline-Val-Asp-difluorophenoxymethylketone (QVD-OPh; HY-12305), and adenosine 5’-triphosphate (HY-B2176; ATP) were from MedChemExpress (Monmouth Junction, NJ, USA). 4′,6-Diamidino-2-phenylindole (DAPI; 14285) and valinomycin were from Cayman Chemical (Ann Arbor, MI, USA). IFNγ (Cat. N° IF002), phorbol 12-myristate 13-acetate (PMA; P8139), and ionomycin calcium salt (I0634s) were from Sigma-Aldrich (St. Louis, MO, USA). Recombinant human sCD40 ligand (sCD40L; 310-02) was from PeproTech (London, UK).

### Cell culture

MEC-1 cells were from Deutsche Sammlung von Mikroorganismen und Zellkulturen GmbH (Braunschweig, Germany) and were maintained in Iscove’s modified Dulbecco’s medium (Gibco; Grand Island, NY, USA) supplemented with 10% heat-inactivated fetal bovine serum, 100 U/mL penicillin, and 100 µg/mL streptomycin. MEC-1 VER cells were established by culturing MEC-1 cells in the presence of venetoclax for 3 months. During the selection process, the cells were maintained in Iscove’s modified Dulbecco’s medium, supplemented with 10% heat-inactivated fetal bovine serum, 100 U/mL penicillin, 100 µg/mL streptomycin, and 100 μg/mL normocin (InvivoGen; San Diego, CA, USA) to prevent contamination with mycoplasma. MEC-1 and MEC-1 VER cells were passaged every 2–3 days and maintained at 0.5–2.0 × 10^6^ cells/mL. To sustain resistance, 10 µM venetoclax was added to the cell cultures on every other passage. Prior to the experiments, MEC-1 VER cells were washed with phosphate-buffered saline (PBS) and resuspended in venetoclax-free and normocin-free fresh culture medium. Primary CLL cells were maintained at 1–2 × 10^6^ cells/mL in Roswell Park Memorial Institute 1640 medium (Cat. N° R5886, Sigma-Aldrich; St. Louis, MO, USA), supplemented with 10% heat-inactivated fetal bovine serum, 2 mM L-glutamine, 100 U/mL penicillin, and 100 µg/mL streptomycin.

All of the cell lines were tested for mycoplasma contamination using the Mycoplasmacheck qPCR test (Eurofins Genomics, Ebersberg, Germany).

### Patient-derived CLL cells

#### Clinical characteristics of patients

The clinical study included 24 patients with immunophenotypically confirmed diagnosis of CLL (15 males, 9 females). At the time of sample collection, one patient was undergoing treatment with venetoclax. The clinical characteristics of the patients are summarized in Table 1.

**Table 1:** Clinical characteristics of the patients with CLL.

Characteristics	Data
Male [n/N (%)]	15/24 (62.5)
Age (years) [mean (range)]	71 (55–92)
Time from diagnosis (years) [mean (range)]	5 (0–21)
Del(13q) [n/N (%)]	9/19 (47.4)
Trisomy 12 [n/N (%)]	6/19 (31.6)
Del(11q) [n/N (%)]	2/19 (10.5)
Del(17p) [n/N (%)]	5/19 (26.3)
Unmutated IGHV [n/N (%)]	8/11 (72.7)
Mutated TP53 [n/N (%)]	0/9 (0)
Treatment [n/N (%)]	10/24 (41.7)

*Del(13q)* deletion of the long arm of chromosome 13, *del(11q)* deletion of the long arm of chromosome 11, *del(17p)* deletion of the short arm of chromosome 17, *IGHV* immunoglobulin heavy chain variable region genes.

#### Isolation by negative selection

Patient-derived CLL cells were isolated from 5 mL of blood obtained from CLL patients, by negatively selecting B-cells with RosetteSep human B-cell enrichment cocktail (STEMCELL Technologies; Serumwerk Bernburg AG, Germany). Briefly, 5 mL of whole blood was centrifuged at 400× *g* for 10 min, then 2–3 mL of plasma was frozen at –80 °C. The amount of plasma removed was substituted with an equal amount of 2% heat-inactivated fetal bovine serum/PBS. The samples were mixed well, and then 100 µL of RosetteSep human B-cell enrichment cocktail was added. Following an incubation at room temperature for 20 min, the samples were diluted with 5 mL of 2% heat-inactivated fetal bovine serum/PBS and carefully layered on 5 mL of density gradient medium (Ficoll Paque Plus; Sigma-Aldrich; St. Louis, MO, USA). Followed centrifugation at 200× *g* for 20 min, brake off. The B-cells were harvested using a pipette and washed with 10 mL culture medium and, where needed, the remaining erythrocytes were lysed with 5 mL of red blood cells lysis buffer (8.02 mg/mL NH_4_Cl, 0.84 mg/mL NaHCO_3_, and 0.37 mg/mL 2,2’,2”,2”‘-(Ethane-1,2-diyldinitrilo)tetraacetic acid, pH 7.4). The purity of the isolated CLL cells was determined using a flow cytometer (Attune NxT; Invitrogen; Carlsbad, CA, USA) and an anti-CD19-PE antibody (Cat. N° 302208; BioLegend; San Diego, CA, USA). The mean (±SD) of the CD19-positivity was 94.2% (±4.3%). Primary CLL cells with poor viability after isolation/thawing were excluded from this study.

### PrestoBlue assay

The PrestoBlue assay is a fluorimetric assay that measures the reduction of blue resazurin to the pink, highly fluorescent resorufin in mitochondria of cells, and can thus be used to assess the metabolic activity of the cells. The assay was carried out as described previously [40]. Briefly, the MEC-1 cells (3 × 10^5^ cells/mL) and primary CLL cells (1 × 10^6^ cells/mL) were left untreated (control) or were treated with 0.1% DMSO (vehicle control) or with the indicated compounds. Then, 100 µL of cell suspension was seeded per well of a black 96-well plate and incubated for the indicated periods at 37 °C in a 5% CO_2_ humidified atmosphere. Then, 10 µL PrestoBlue reagent (Invitrogen, Carlsbad, CA, USA) was added to each well, and the plate was incubated at 37 °C for 1–3 h. The fluorescence intensity of resorufin (I) was measured using a microplate reader (Synergy HTX Multi-Mode Microplate Reader; BioTek Instruments, Inc., Winooski, VT, USA) with excitation/emission filters of 530/590 nm. The relative metabolic activities were calculated as (I_sample_ − I_blank_)/(I_control_ − I_blank_) × 100 (%). The data are means ± SEM of ≥three independent experiments, each carried out in duplicate.

### Cytotoxicity assay with propidium iodide

MEC-1 and MEC-1 VER cells (3 × 10^5^ cells/mL) were treated with 0.1% DMSO (vehicle control) or 1–50 µM venetoclax. Then, 100 µL of cell suspension was added in a duplicate to each well of a 96 well plate and incubated for the indicated periods (37 °C, 5% CO_2_, humidified atmosphere). After that, 5 µM propidium iodide (PI; Molecular Probes, Eugene, OR, USA) was added and incubated for 5 min with a gentle shaking of the plate. Afterward, a minimum of 10,000 events was collected using an autosampler connected to a flow cytometer (Attune NxT; Invitrogen; Carlsbad, CA, USA). Data are means ± SEM of three independent experiments, each carried out in duplicate.

### SYTOX Blue/annexin V assay

Detection of early apoptosis with SYTOX Blue/annexin V assay was carried out as described previously [40]. Briefly, CLL cells (1 × 10^6^ cells/mL) and MEC-1 cells (3 × 10^5^ cells/mL) were treated with 0.1% DMSO or with the compounds of interest for 24 h and then harvested for determination of phosphatidylserine exposure using R-phycoerythrin conjugated annexin V (R-PE Annexin V; Invitrogen; Carlsbad, CA, USA) and membrane integrity using the nucleic acid stain SYTOX Blue dead cell stain (Invitrogen; Carlsbad, CA, USA). The cells were washed with 0.3 mL ice-cold PBS and resuspended in annexin-binding buffer at 1 × 10^6^ cells/mL. To each 100 µL of sample, 2.5 µL annexin V was added, incubated in the dark for 15 min, and then 200 µL annexin-binding buffer with SYTOX Blue was added (final concentration, 750 nM). A minimum of 10,000 events per sample was collected using an autosampler connected to a flow cytometer (Attune NxT; Invitrogen; Carlsbad, CA, USA). The experiment was repeated three times, representative dot plots are shown. Cells that stained double negative for annexin V and SYTOX Blue (ANV-/SB-) were not undergoing measurable apoptosis and were thus considered viable. Conversely, cells that stained double-positive for annexin V and SYTOX Blue (ANV + /SB + ) were considered as late-apoptotic or dead. The cells that stained positive for annexin V (ANV + /SB-) only were undergoing early apoptosis, while those that stained positive for SYTOX Blue only were considered necrotic. Data are means ± SEM of three independent experiments.

### Determination of the mitochondrial membrane potential

The mitochondrial membrane potential (ΔΨm) was determined using MitoProbe JC-1 Assay Kit (Invitrogen; Carlsbad, CA, USA). MEC-1 cells (3 × 10^5^ cells/mL) were treated with 0.1% DMSO or 100 nM, 250 nM, 500 nM ONX-0914 for 24 h. For positive control, cells were treated with 1 µM valinomycin for 1 h. Then, cells were pelleted (200× *g*, 5 min), resuspended in 300 µL PBS with 2 µM JC-1, and incubated in the dark for 20 min. After a wash with 1 mL PBS (200× *g*, 5 min), cells were resuspended in 500 µL PBS and the intensity of red (high ΔΨm) and green (low ΔΨm) fluorescence of JC-1 was determined using flow cytometry. A minimum of 10,000 events was collected using a flow cytometer (Attune NxT; Invitrogen; Carlsbad, CA, USA). Data are means ± SEM of three independent experiments.

### Nuclear translocation of NFκB

Patient-derived CLL cells (2 × 10^6^ cells/mL) were seeded in a 12 well plate and incubated with 0.1% DMSO, 100 ng/mL IFNγ, 10 nM PMA/ 1 µM ionomycin, and 100 ng/mL sCD40L for 1 h at 37 °C, in a 5% CO_2_ humidified atmosphere. The samples were then centrifuged (300× *g*, 5 min), the supernatants were discarded, and the cells were fixed in 200 µL 4% paraformaldehyde for 10 min. PBS (1.4 mL) was added to each of the samples, which were then vortexed and centrifuged (300× *g*, 5 min). The cells were resuspended in 100 µL NFκB p65 (D14E12) XP rabbit mAb (#8242; Cell Signaling Technology; Danvers, MA, USA), prepared as a 1:1,000 dilution in 0.1% Triton X-100/5% bovine serum albumin/PBS, and incubated at room temperature for 20 min. Afterward, the cells were washed by addition of 1.5 mL PBS, and then resuspended in 100 µL 0.1% Triton X-100/5% bovine serum albumin/PBS containing 3 µM DAPI and anti-rabbit IgG (H+L), F(ab’)2 fragment (Alexa Fluor 647 conjugate) (#4414; Cell Signaling Technology; Danvers, MA, USA) at a 1:1,000 dilution. After a 15-min incubation at room temperature (in the dark), the samples were washed by adding 1.5 mL PBS, which was followed by another wash with 1.5 mL PBS. The cell pellet was then resuspended in 25 µL PBS and a minimum of 5,000 events in focus was collected per sample using an imaging flow cytometer (Amnis ImageStream X Mk II; Luminex Corporation; Austin, TX, USA). Translocation of p65 was evaluated using the built-in protocol Nuclear Localization (IDEAS). The image channels Ch06, Ch07, and Ch11 were used for brightfield, nuclear image (DAPI), and the translocating probe (p65), respectively. Gating strategy: events with a focused nuclear image (normalized frequency vs gradient RMS_M07_Ch07), followed by gating single cells (aspect ratio_M06 vs area_M06), and then DAPI and p65 double-positive events (intensity_MC_Ch11 vs intensity_MC_Ch07). These events were selected to obtain the proportion of cells that were translocating NFκB to the nucleus (normalized frequency vs similarity_morphology (M07, Ch07)_Ch11_Ch07). The data are means ± SEM of three independent experiments.

### Immunoblotting

Immunoblotting was performed as described previously. Samples from MEC-1 and MEC-1 VER cells were prepared by culturing 2–4 mL of cells (1 × 10^6^ cells/mL) in growth medium, supplemented with 2% heat-inactivated fetal bovine serum overnight. These cells were used to define the levels of proteins under basal and stimulated conditions. Samples from primary CLL cells were prepared by culturing 2–4 mL of cells (2 × 10^6^ cells/mL) in growth medium, supplemented with 2% heat-inactivated fetal bovine serum. The cells were centrifuged (400× *g*, 5 min, 4 °C), washed with 1 mL cold PBS (400× *g*, 5 min, 4 °C), and lysed in 100 µL modified RIPA buffer (50 mM Tris-HCl, 150 mM NaCl, 0.5 % sodium deoxycholate, 1 mM EDTA, 1% Nonidet P-40, pH = 7.4), containing 1 µL Halt Protease Inhibitor Cocktail (100x) (Cat. N° 87785; Thermo Fisher Scientific, Waltham, MA, USA) and 1 µL Halt Phosphatase Inhibitor Cocktail (100x) (Cat. N° 78420; Thermo Fisher Scientific; Waltham, MA, USA), and frozen at –80 °C. The proteins were isolated from the cells by first thawing the samples on ice. Then, the samples were sonicated twice for 8 s, vortexed, and then centrifuged at 10,000× *g* for 20 min at 4 °C. The supernatants were collected and the total protein concentration was determined using the DC method. SDS-PAGE was used for the separation of proteins. The samples were mixed with 33% sample loading buffer (10% glycerol, 0.1% bromophenol blue, 3% sodium dodecyl sulfate (SDS)) and 5% 2-mercaptoethanol, incubated at 95 °C for 5 min, and cooled on ice. Then 25 µg of proteins was separated on 10% gels, using Tris-glycine electrophoresis buffer (25 mM Tris-base, 192 mM glycine, 0.1% SDS, pH = 8.3) and constant voltage (110 V) for 90 min. Afterward, the proteins were blotted to 0.4 µm nitrocellulose membrane using ice-cold transfer buffer (20% methanol, 25 mM Tris-base, 192 mM glycine, 0.1% SDS, pH = 8.3) and constant voltage (100 V) for 60 min. The membranes were blocked in 5% milk/Tris-buffered saline (TBS; 25 mM Tris-base, 137 mM NaCl, 3 mM KCl, pH = 7.4) with 0.1% Tween 20 (TTBS) for 1 h, washed with TTBS (3× 5 min), and then incubated with the primary antibodies (1:1,000 dilution in 5% bovine serum albumin/TTBS) overnight at 4 °C. Then, the membranes were washed with TTBS (5× 5 min) and incubated with the appropriate secondary antibody (1:10,000 dilution in 5% bovine serum albumin/TTBS) for 1 h at room temperature. Following the final wash, 200–500 µL SuperSignal West Femto Maximum Sensitivity Substrate (Cat. N° 34095; Thermo Fisher Scientific; Pierce, IL, USA) was added to the membrane and the chemiluminescence was measured with a chemiluminescence imaging system (Uvitec Cambridge Alliance 9.7; Uvitec; Lodi, NJ, USA). For the loading control, the membranes were stripped (62.5 mM Tris/HCl, 2% SDS, 100 mM 2-mercaptoethanol, pH = 6.8; 30 min at 50 °C) and reprobed with an antibody against β-actin (1:5,000 dilution; A5316; Sigma-Aldrich; St. Louis, MO, USA), α/β-tubulin (1:1,000 dilution; #2148; Cell Signaling Technology; Danvers, MA, USA) or GAPDH (1:1000 dilution; #97166; Cell Signaling Technology, Danvers, MA, USA). The primary antibodies against p38 (#9212), p-p38 MAPK (Thr180/Tyr182) (#9215), JNK (#9252), p-JNK (Thr183/Tyr185) (#9251), MEK1/2 (#8727), p-MEK1/2 (Ser217/221) (#9154), p-ERK1/2 (Thr202/Tyr204) (#9101), CREB (#9197), p-CREB (Ser133) (#9191), Bcl-2 (#15071), p-Bcl-2 (#2827) Bcl-xL (#2764), Bid (#2002), Puma (#12450), Bax (#2772), Bak (#12105), and Mcl-1 (#94296) were from Cell Signaling Technology (Danvers, MA, USA). The antibodies against cyclin D1 (sc-718), ERK1 (sc-93), and ERK2 (sc-154; 1:5,000) were from Santa Cruz Biotechnology (Dallas, TX, USA). The secondary antibodies were horse anti-mouse IgG, HRP-linked antibody (#7076; Cell Signaling Technology; Danvers, MA, USA) or goat anti-rabbit IgG antibody, HRP-conjugate (12-348; EMD Millipore; Burlington, MA, USA). Data are means ± SEM of ≥ two independent experiments. Full and uncropped immunoblots are presented in Supplemental File.

### Activity of the immunoproteasome in whole-cell lysates

To assess the catalytic activities of β1i (LMP2), β2/β2i (β2/MECL1), and β5i (LMP7), the following substrates were used: Ac-Pro-Ala-Leu-AMC (Ac-PAL-AMC; 25 µM; South Bay Bio; San Jose, CA, USA), Ac-RLR-AMC (50 µM; Cayman Chemical; Ann Arbor, MI, USA), and Ac-Ala-Asn-Trp-AMC (Ac-ANW-AMC; 25 µM; South Bay Bio; San Jose, CA, USA), respectively. The levels of nonspecific hydrolysis of the substrates were determined using selective inhibitors: 1000 nM ML604440 for LMP2; 100 nM M3258 for LMP7; and 1000 nM marizomib (pan-proteasome inhibitor) for β2/β2i (β2/MECL1). The residual activities were <10%. MEC-1 cells or patient-derived CLL cells (1 × 10^6^ cells/mL, 4 mL) were treated with 0.1% DMSO and 100 ng/mL IFNγ as indicated. The cells were then centrifuged (300× *g*, 5 min) and washed with 1 mL PBS (300× *g*, 5 min), and the cell pellets were lysed in 100 µL lysis buffer (50 mM Tris-HCl, 0.5 mM EDTA, pH = 8.0). The isolation of proteins and the determination of total protein concentration was performed as described above. Following addition of 10 µL whole-cell lysate to 80 µL assay buffer (5 mM dithiothreitol, 2 mM adenosine 5’-triphosphate, 50 mM Tris-HCl, 0.5 mM EDTA, pH 8.0) without and with the appropriate inhibitors in each well of black 96-well plates, the samples were incubated for 30 min at 37 °C. Then 10 µL of a substrate prepared in assay buffer was added, and the release of AMC was measured over 1 h at 1-min intervals using a microplate reader (Synergy HTX Multi-Mode Microplate Reader; BioTek Instruments; Inc., Winooski, VT, USA), with excitation/emission filters of 360/460 nm. The fluorescence intensities (I) were used to calculate relative enzyme activity, using the following equation: (I_sample_ − I_blank_)/(I_control_ − I_blank_). The data were normalized to total protein concentration. Data are means ± SEM of ≥ three independent experiments, each carried out in duplicate.

### Whole-exome sequencing

Whole-exome sequencing of MEC-1 and MEC-1 VER cells was performed by NGS Lab Constance

Eurofins Genomics Europe Sequencing GmbH (Constance, Germany), using Genome Sequencer Illumina NovaSeq, sequencing mode NovaSeq 6000 S4 PE150 XP. Data are registered with BioProject (ID: PRJNA873921); BioSample (ID: SUB11975361) accessions are SAMN30508221 and SAMN30508222 for MEC-1 and MEC-1 VER samples, respectively.

### Statistical analysis

Data obtained with flow cytometer were processed using the FlowJo (version 10.7.2) software, and the data obtained with the imaging flow cytometer were processed using the IDEAS (version 6.2) software. Data presentation and statistical analysis were performed in GraphPad Prism 9.2.0 using the following statistical tests: Student t-test (two-sided), one-way ANOVA, and two-way ANOVA. Statistical significance: *p* > 0.05 denotes not significant (ns); Asterisks *, **, ***, and **** denote *p* < 0.05, *p* < 0.01, *p* < 0.001, and *p* < 0.0001, respectively.

## Results

### IFNγ rescues CLL cells from spontaneous and venetoclax-induced cell death and activates the immunoproteasome

**Fig. 1:**
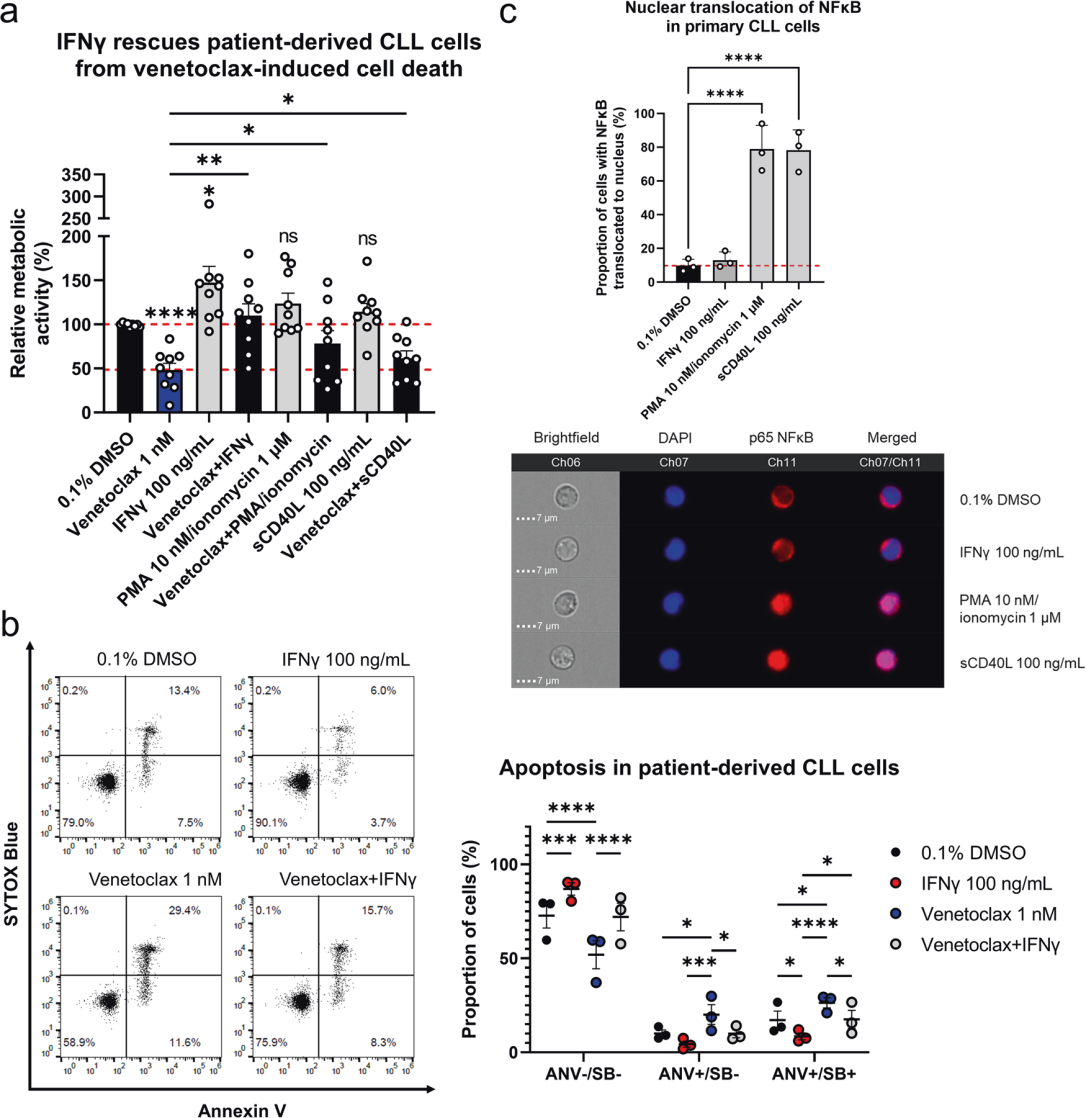
IFNγ rescues CLL cells from spontaneous and venetoclax-induced cell death. **a** Diverse stimulants abrogate venetoclax cytotoxicity against patient-derived CLL cells. Primary cells (1 × 10^6^ cells/mL) obtained from nine patients with CLL were treated with 0.1% DMSO (vehicle control), and 1 nM venetoclax alone and in combination with 100 ng/mL IFNγ, 10 nM PMA/1 µM ionomycin, and 100 ng/mL sCD40L for 24 h. The metabolic activities of the cells were determined using the PrestoBlue assay. Data are means ± SEM of ≥ three independent experiments, each carried out in duplicate. Two-way ANOVA (post hoc Tukey); *, **, and **** denote *p* < 0.05, *p* < 0.01, and *p* < 0.0001, respectively; **b** Cytoprotective effects of IFNγ in the patient-derived CLL cells undergoing spontaneous and venetoclax-induced apoptosis. CLL cells (1 × 10^6^ cells/mL) were treated with 0.1% DMSO (vehicle control) and 1 nM venetoclax, 100 ng/mL IFNγ, and their combination for 24 h. Apoptosis was assessed using the SYTOX Blue/annexin V assay. Representative dot plots (left) and means ± SEM of three independent experiments (right) are shown. Two-way ANOVA (post hoc Tukey); *, **, ***, and **** denote *p* < 0.05, *p* < 0.01, *p* < 0.001, and *p* < 0.0001, respectively; **c** Nuclear translocation of NFκB in the primary CLL cells. CLL cells (1 × 10^6^ **c**ells/mL) from three patients with CLL were treated with 0.1% DMSO (vehicle control), 100 ng/mL IFNγ, 10 nM PMA/1 µM ionomycin, and 100 ng/mL sCD40L for 1 h. Quantification (left) and representative images (right) are shown for the translocation of NFκB to the nucleus, using imaging flow cytometry. One-way ANOVA (post hoc Dunnett); **** denotes *p* < 0.0001.

The tumor microenvironment is an important player in the maintenance and therapeutic susceptibility of CLL cells in vivo. To determine how the activation of CLL cells within the tumor microenvironment affects the cytotoxic effect of venetoclax, we treated cells derived from nine patients with CLL with 1 nM venetoclax alone and in combination with 100 ng/mL IFNγ, 10 nM PMA/ 1 µM ionomycin, and 100 ng/mL sCD40L for 24 h. The metabolic activities of the cells were determined using the PrestoBlue assay. Here, IFNγ, PMA/ionomycin, and sCD40L increased the metabolic activities of the CLL cells by 47%, 24%, and 14%, respectively (Fig. 1a). The metabolic activity of the CLL cells treated with 1 nM venetoclax alone was 48%, which was significantly lower than for the cells co-treated with IFNγ (110%), PMA/ionomycin (78%), and sCD40L (62%). The IFNγ activated CLL cells most prominently and demonstrated exceptional cytoprotective properties against venetoclax, surpassing even BCR- (PMA/ionomycin) and CD40L-mediated cytoprotection.

We next investigated the mechanism of cytoprotection against venetoclax. The primary CLL cells were treated with 0.1% DMSO (vehicle control) and 100 ng/mL IFNγ, 1 nM venetoclax, and their combination for 24 h. Then, the level of apoptosis was determined with the SYTOX Blue/annexin V assay. The treatment of CLL cells with IFNγ rescued the CLL cells from spontaneous and venetoclax-induced apoptosis (Fig. 1b). The viabilities of the cells treated with 0.1% DMSO, IFNγ, venetoclax, and their combination were 72.3%, 87.0%, 51.9%, and 72.0%, respectively. Compared to the vehicle control and the venetoclax-treated cells, the proportion of late apoptotic cells was lower for both the IFNγ-treated cells (8.5% vs 17.1% for vehicle control) and IFNγ co-treated cells (17.5% vs 26.3% for venetoclax-treated cells). Overall, these data indicate that IFNγ-mediated signaling might be an even more important mechanism of resistance of CLL cells to venetoclax than signaling through CD40 and BCR [18, 31].

As NFκB was shown to be the central component of BCR- and CD40L-mediated resistance to venetoclax, we tested whether the same holds true for IFNγ. For this purpose, CLL cells from three different patients with CLL, were treated with 0.1% DMSO (vehicle control), 100 ng/mL IFNγ, 10 nM PMA/1 µM ionomycin, and 100 ng/mL sCD40L for 1 h. Nuclear translocation of p65 (NFκB) was assessed using imaging flow cytometry. Treatment of the CLL cells with PMA/ionomycin and sCD40L triggered translocation in ~80% of the cells, while IFNγ had no effect (Fig. 1c). This showed that IFNγ did not activate the NFκB pathway, thus suggesting that other mechanisms are responsible for this resistance to venetoclax.

**Fig. 2:**
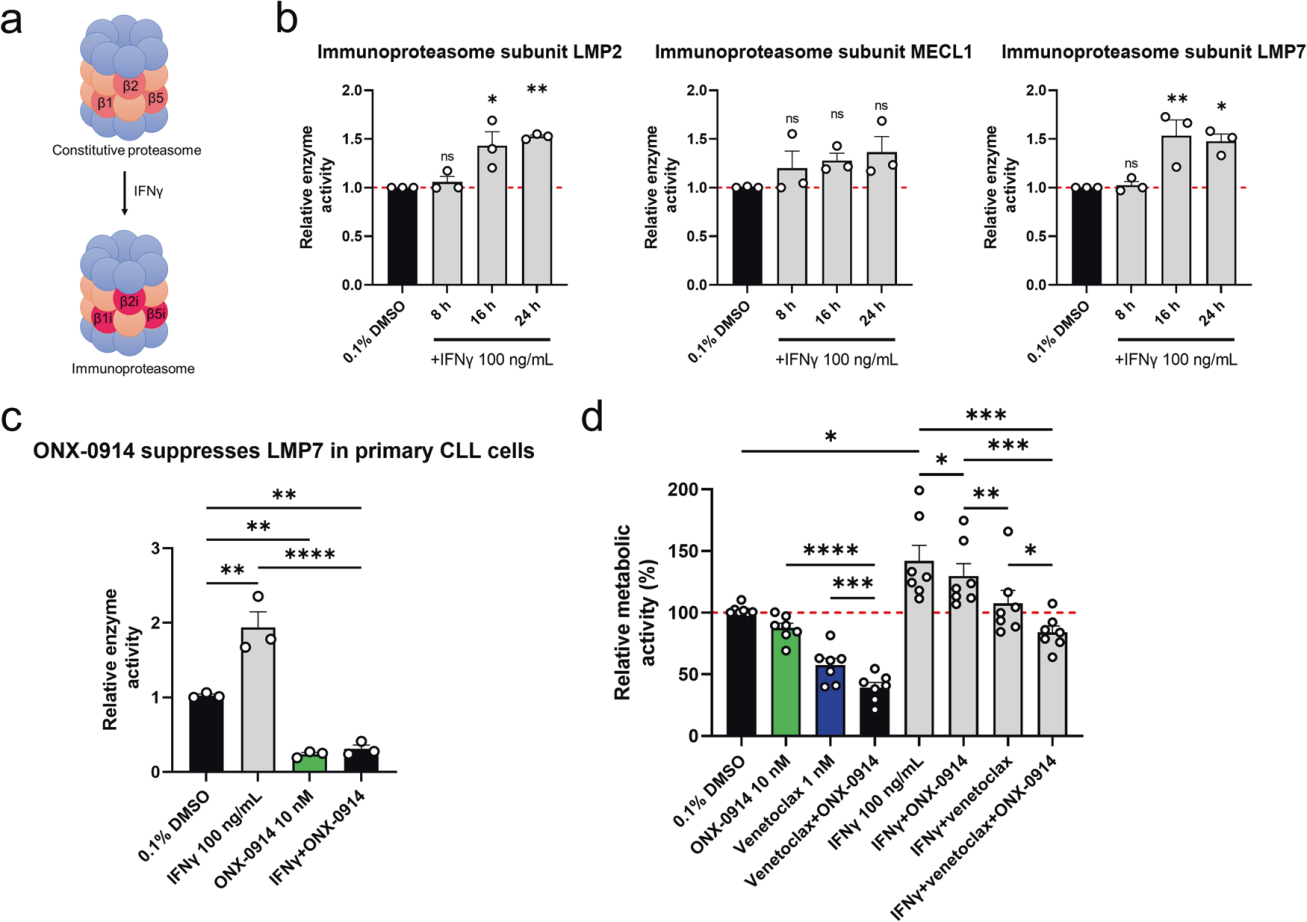
IFNγ activates the immunoproteasome in CLL cells. **a** Schematic depiction of immunoproteasome formation in the presence of IFNγ; **b** MEC-1 cells (1 × 10^6^ cells/mL) were treated with 0.1% DMSO (vehicle control) or 100 ng/mL IFNγ for 8 h, 16 h, and 24 h. The cells (4 × 10^6^) were harvested and lysed, and the activities of LMP2, MECL1, and LMP7 were determined in whole-cell lysates using subunit-specific substrates. Fluorescence of the cleaved substrates at 20 min was normalized to the total protein concentration. Data are means ± SEM of ≥ three independent experiments, each carried out in duplicate. One-way ANOVA (post hoc Dunnett); not significant (ns), * denotes *p* < 0.05; **c** ONX-0914 suppresses basal and IFNγ-induced activity of immunoproteasome subunit LMP7 in primary CLL cells. Cells derived from three patient with CLL (1 × 10^6^ cells/mL) were treated with 0.1% DMSO (vehicle control) and 10 nM ONX-0914, 100 ng/mL IFNγ, and their combination for 24 h. The activity of LMP7 was determined using subunit-specific substrate. Fluorescence of the cleaved substrates at 60 min was normalized to the total protein concentration. Data are means ± SEM of ≥ three independent experiments, each carried out in duplicate. One-way ANOVA (post hoc Dunnett); ** and **** denote *p* < 0.01 and *p* < 0.0001; **d** ONX-0914 synergizes with venetoclax against primary CLL cells. Cells derived from seven patients with CLL (1 × 10^6^ cells/mL) were treated with 0.1% DMSO (vehicle control), 10 nM ONX-0914, 1 nM venetoclax, and their combination in the absence and presence of 100 ng/mL IFNγ for 24 h. Then the metabolic activities of cells were determined using the PrestoBlue assay. Data are means ± SEM of ≥ three independent experiments, each carried out in duplicate. Two-way ANOVA (post hoc Tukey); *, **, ***, and **** denote *p* < 0.05, < 0.01, < 0.001, and *p* < 0.0001.

In immune cells and in the presence of proinflammatory cytokines (e.g., IFNγ), the catalytically active proteasome subunits β1, β2, and β5 are replaced by β1i (LMP2), β2i (MECL1), and β5i (LMP7), respectively, to form the immunoproteasome (Fig. 2a) [21, 22]. Thus, we postulated that IFNγ increases the activity of the immunoproteasome. To test this, MEC-1 cells were treated with 0.1% DMSO (vehicle control) or 100 ng/mL IFNγ for 8 h, 16 h, and 24 h. Afterward, the cells were harvested and lysed, and the activity of the immunoproteasome was determined by measuring the hydrolysis of subunit-specific substrates. The activities of immunoproteasome subunits LMP2 and LMP7 increased in a time-dependent manner, while the activity of β2/MECL1 did not change significantly (Fig. 2b). This indicates that the majority of the immunoproteasome catalytic activity comes from LMP2 and LMP7, defining their importance for IFNγ-mediated resistance to venetoclax, and as such, as potential targets in the treatment of CLL.

To corroborate this further, we next tested, whether the inhibitor of immunoproteasome subunit LMP7 ONX-0914 suppresses the activity of immunoproteasome in patient-derived cells. Primary cells of three patients with CLL were treated with 0.1% DMSO (vehicle control), 10 nM ONX-0914, 100 ng/mL IFNγ, and their combination for 24 h. Then, the samples were lysed and the activity of immunoproteasome subunit LMP7 was assessed using subunit-specific substrate. We showed that IFNγ increased the activity of LMP7 in primary samples to 194% of vehicle control, while ONX-0914 efficiently suppressed basal and IFNγ-induced activity of LMP7 to 24% and 31%, respectively (Fig. 2c). We next proposed that ONX-0914 overcomes IFNγ-mediated resistance of patient-derived CLL cells to venetoclax. To test this, we treated cells derived from seven CLL patients with 0.1% DMSO (vehicle control), 10 nM ONX-0914, 1 nM venetoclax, and their combination alone and in combination with 100 ng/mL IFNγ for 24 h. Then, the metabolic activities of the cells were determined using the PrestoBlue assay. We demonstrated that ONX-0914 synergized with venetoclax against CLL cells (Fig. 2d). The mean metabolic activity of cells treated with ONX-0914 and venetoclax was 88% and 57%, and it decreased further to 39% for cells treated with the combination, thus demonstrating that ONX-0914 enhances the cytototoxicity of venetoclax. On the other hand, IFNγ rescued CLL cells from venetoclax cytotoxicity, which was counteracted by ONX-0914 (Fig. 2d). The metabolic activitiy of IFNγ-primed CLL cells was 142% of vehicle control and the treatment with 10 nM ONX-0914 decreased this to 130%. IFNγ-primed cells were not affected by venetoclax (108%), but the addition of 10 nM ONX-0914 to venetoclax decreased the metabolic activity of cells to 84%. Collectively, these data demonstrate that ONX-0914 can tackle venetoclax resistance in CLL.

### Establishment of an in-vitro model of venetoclax-resistant CLL

**Fig. 3:**
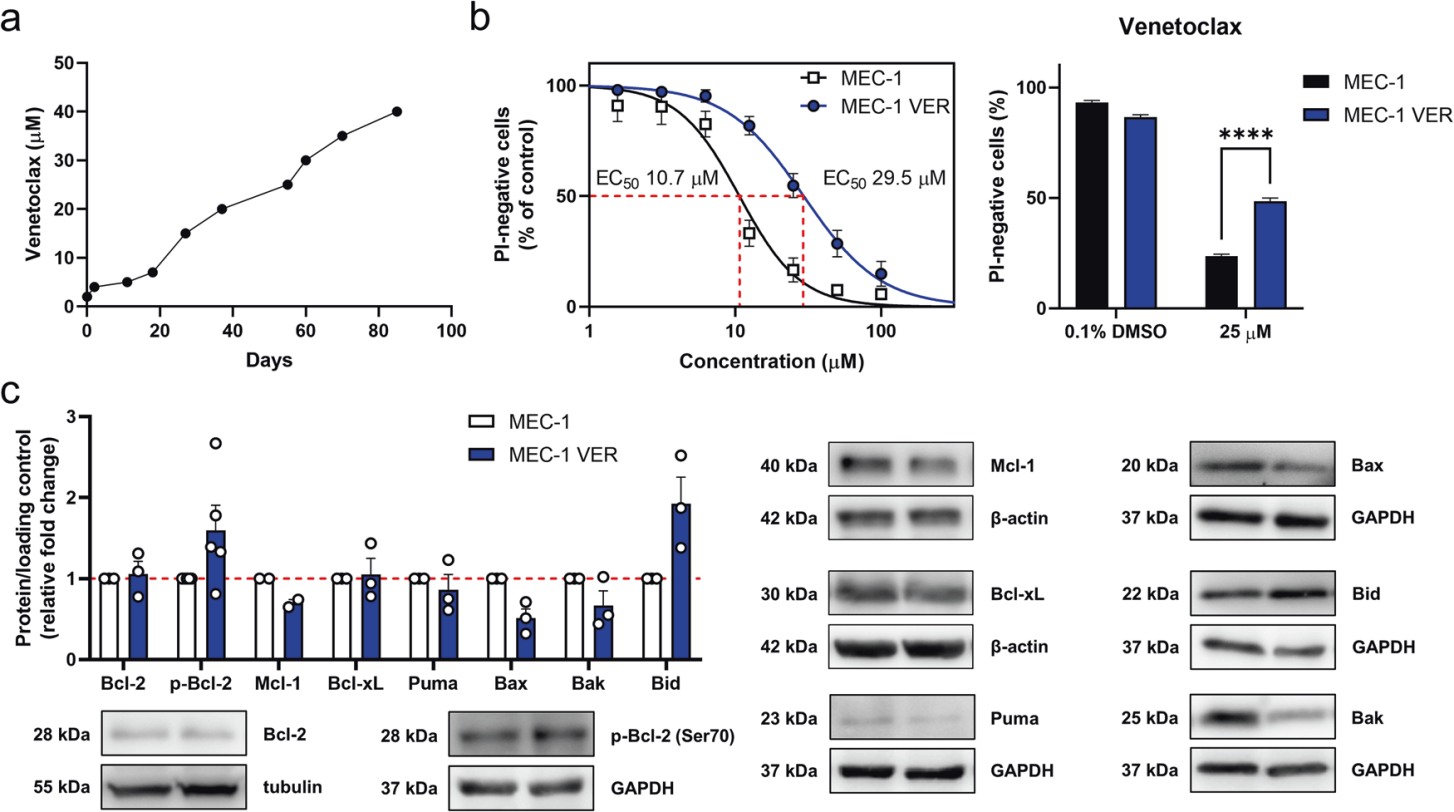
Establishment of venetoclax-resistant MEC-1 VER cells. **a** Dosing regimen used for the selection of venetoclax-resistant clones. MEC-1 cells (1 × 10^6^ cells/mL) were seeded into a 12 well plate and incubated with gradually increasing concentrations of venetoclax, from 1 µM at day 0, to 40 µM at day 85; **b** Cytotoxicity of venetoclax in MEC-1 and MEC-1 VER cells. The cells (3 × 10^5^ cells/mL) were treated with 0.1% DMSO (vehicle control) and venetoclax (1–100 µM) for 24 h. Afterward, 5 µM PI was added and the viability of cells was determined using flow cytometry. EC_50_ values were calculated using GraphPad Prism 9.2.0 (left). The viabilities of these MEC-1 and MEC-1 VER cells upon treatment with 25 µM venetoclax for 24 h are also shown (right). Data are means ± SEM of three independent experiments, each carried out in duplicate. Statistical significance was determined using paired Student t-test; **** denotes *p* < 0.0001; **c** Expression of anti-apoptotic proteins in MEC-1 and MEC-1 VER cells. Whole-cell lysates of MEC-1 and MEC-1 VER cells, cultured under basal conditions, were probed for Bcl-2, p-Bcl-2 (Ser70), Mcl-1, Bcl-xL, Puma, Bax, Bak, and Bid levels using immunoblotting. Data are means ± SEM of three independent experiments. Representative immunoblots are also shown.

To study the resistance of CLL cells to venetoclax further, we established an in-vitro model of venetoclax resistant CLL cells by culturing MEC-1 cells in the presence of gradually increasing concentrations of venetoclax for 3 months (Fig. 3a). These clonally selected cells were termed MEC-1 VER (VE, venetoclax; R, resistant) cells.

The resistance to venetoclax of these *de-novo* established MEC-1 VER cells was confirmed by determination of the EC_50_ of venetoclax, using flow cytometry. Compared to MEC-1 cells, the MEC-1 VER cells showed an EC_50_ for venetoclax that was nearly 3-fold higher (10.7 vs. 29.5 μM, respectively; Fig. 3b). Here, the treatment of these MEC-1 and MEC-1 VER cells with 25 µM venetoclax for 24 h reduced their viabilities to 24% and 48%, respectively (Fig. 3b).

Immunoblotting was then used to investigate the anti-apoptotic proteins Bcl-2, Mcl-1, and Bcl-xL, which are normally responsible for venetoclax resistance in B-cell malignancies [41], and proapoptotic proteins Puma, Bax, Bak, and Bid. These data show that the total levels of Bcl-2 and Bcl-xL did not differ between the MEC-1 and MEC-1 VER cells. While the levels of Mcl-1, Bax, and Bak were lower by 30%, 49%, and 33%, respectively, in MEC-1 VER cells (Fig. 3c), the levels of p-Bcl-2 (Ser70) and Bid were higher by 60% and 92%, respectively. This proposes that MEC-1 VER cells adapted to venetoclax by decreasing proapoptotic effectors of Bcl-2 family and more importantly highlights the phosphorylation of Bcl-2 at Ser70 as the resistance-driving mechanism in these cells [42]. Whole-exome sequencing confirmed that MEC-1 VER cells do not harbor resistance-driving mutations in the BCL2 gene.

### Inhibition of immunoproteasome overcomes resistance of MEC-1 VER cells to venetoclax and induces apoptotic cell death

**Fig. 4:**
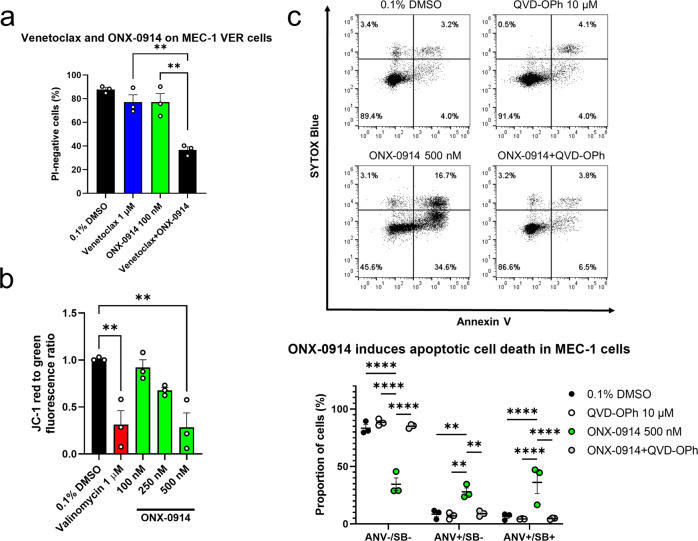
Immunoproteasome inhibitor ONX-0914 overcomes resistance of MEC-1 VER cells to venetoclax. **a** MEC-1 VER cells (3 × 10^5^ cells/mL) were treated with 0.1% DMSO (vehicle control), 1 µM venetoclax, 100 nM ONX-0914 and their combination for 24 h. Then, the samples were stained with 5 µM propidium iodide (PI) and analyzed using flow cytometry. Data are means ± SEM of three independent experiments, each carried out in duplicate. One-way ANOVA (post hoc Dunnett); ** denotes *p* < 0.01. **b** ONX-0914 disrupts mitochondrial membrane potential. MEC-1 cells (3 × 10^5^ cells/mL) were treated with 0.1% DMSO or 100, 250, and 500 nM ONX-0914 for 24 h and with 1 µM valinomycin (positive control) for 1 h. Then, the samples were stained with JC-1 probe and analyzed using flow cytometry. Data are means ± SEM of three independent experiments. One-way ANOVA (post hoc Dunnett); * denotes p < 0.05. **c** ONX-0914 induces caspase-dependent apoptotic cell death. MEC-1 cells (3 × 10^5^ cells/mL) were treated with 0.1% DMSO, 500 nM ONX-0914, 10 µM pan-caspase inhibitor QVD-OPh and their combination for 24 h. The proportion of cells undergoing early (lower right quadrant; ANV + /SB-) and late (upper right quadrant; ANV + /SB + ) apoptosis was determined. Representative dot plots (above) and means ± SEM of three independent experiments (below) are shown. Two-way ANOVA (post hoc Tukey); **, and **** denote *p* < 0.05 and *p* < 0.0001, respectively.

We next tested whether pharmacological inhibition of immunoproteasome with selective inhibitor ONX-0914 is able to overcome resistance of these MEC-1 VER cells to venetoclax. MEC-1 VER cells were treated with 0.1% DMSO (vehicle control), 1 µM venetoclax, 100 nM ONX-0914 and their combination for 24 h. Then, the cell viability was determined using flow cytometry. ONX-0914 resensitized MEC-1 VER cells to venetoclax (Fig. 4a). The viability of MEC-1 VER cells treated with venetoclax and ONX-0914 was 77% and it decreased to 37% for cells treated with combination. This demonstrates that ONX-0914 overcomes resistance of MEC-1 VER cells to venetoclax. To gain an insight into the cytotoxic action of ONX-0914, we analyzed hallmarks of apoptotic cell death, that is disruption of ΔΨm, exposure of phosphatidylserine, and involvement of caspases following the 24-h treatment of MEC-1 cells with ONX-0914 (Fig. 4b,c). ONX-0914 induced concentration-dependent decrease in the red-to-green ratio of JC-1 probe, thus demonstrating that it disrupts ΔΨm. We next treated MEC-1 cells with 0.1% DMSO (vehicle control), 500 nM ONX-0914, 10 µM pan-caspase inhibitor QVD-OPh and their combination for 24 h. Externalization of phospatidylserine was then assessed using annexin V and flow cytometry. The treatment of MEC-1 cells with ONX-0914 increased the proportion of early apoptotic (28.0% vs 8.5% for vehicle control) and late apoptotic cells (36.2% vs 6.5% for vehicle control). The addition of QVD-OPh to ONX-0914-treated cells rescued MEC-1 cells from cell death, demonstrating that ONX-0914 induces caspase-dependent cell death. Collectively, these data demonstrate that selective immunoproteasome inhibition induces intrinsic apoptosis in CLL cells.

### Inhibition of p38 MAPK abrogates the cytoprotective effects of IFNγ

We next investigated, whether the activating and cytoprotective functions of IFNγ can be overturned to prevent relapses of patients with CLL treated with venetoclax. We postulated that inhibition of the p38 MAPK pathway can counteract the effects of IFNγ. To test this, cells derived from 10 patients with CLL were treated with 100 ng/mL IFNγ alone and in combination with 50 µM BIRB796 or 50 µM SB203580 for 24 h.

**Fig. 5:**
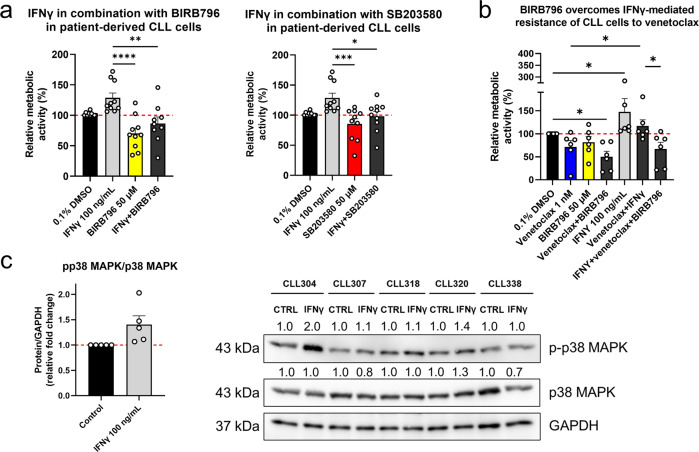
Inhibition of p38 MAPK can reverse the cytoprotective effects of IFNγ. **a** Block on p38 MAPK pathway abolishes the stimulating effects of IFNγ in CLL cells. Primary cells (1 × 10^6^ cells/mL) obtained from 10 patients with CLL were treated with 0.1% DMSO (vehicle control) and 100 ng/mL IFNγ alone and in combination with 50 µM BIRB796 or 50 µM SB203580 for 24 h. The metabolic activity of cells was determined using the PrestoBlue assay. Data are means ± SEM of ≥ three independent experiments, carried out in duplicate. One-way ANOVA (post hoc Dunnett); *, **, ***, and **** denote p < 0.05, *p* < 0.01, *p* < 0.001, and *p* < 0.0001; **b** Inhibition of p38 MAPK reverses IFNγ-mediated resistance of primary CLL cells to venetoclax. CLL cells (1 × 10^6^ cells/mL) from six patients with CLL were treated with 0.1% DMSO (vehicle control), 1 nM venetoclax, 50 µM BIRB796, 100 ng/mL IFNγ, and their combinations for 24 h. The metabolic activity of cells was determined using the PrestoBlue assay. Data are means ± SEM of ≥ three independent experiments, each carried out in duplicate. Two-way ANOVA (post hoc Tukey); *, denotes *p* < 0.05; **c** IFNγ activates the p38 MAPK pathway in primary CLL **c**ells. Cells (2 × 10^6^ cells/mL) from five patient with CLL were left untreated (control) or were treated with 100 ng/mL IFNγ for 1 h. Then, the cells were lysed, and whole cells lysates were separated with SDS-PAGE and blotted to nitrocellulose membranes. The ratio p-p38 MAPK/p38 MAPK (left) and immunoblots (right) for phosphorylated and total forms of p38 MAPK are shown. GAPDH was used as the loading control. Data are means ± SEM of five independent experiments.

Here, both of these p38 MAPK inhibitors abolished the stimulating effects of IFNγ on these patient-derived CLL cells (Fig. 5a). The metabolic activity of the cells treated with IFNγ was 129% and was reduced to 87% and 98% for cells co-treated with BIRB796 and SB203580, respectively. Next, we investigated, whether BIRB796 can overcome the IFNγ-mediated resistance of the CLL cells to venetoclax. We treated cells derived from six patients with CLL with 0.1% DMSO (vehicle control), 50 µM BIRB796, 100 ng/mL IFNγ, 1 nM venetoclax, and the indicated combinations for 24 h (Fig. 5b). IFNγ again rescued the CLL cells from the effects of venetoclax. However, more importantly, the addition of BIRB796 to the combination of venetoclax and IFNγ restored the cytotoxicity of venetoclax. The metabolic activity of the cells treated with the combination of venetoclax and IFNγ was 117%, which was decreased to 67% upon addition of BIRB796. This was comparable to the metabolic activity of the cells treated with venetoclax and BIRB796 (50%). Overall these data confirm that inhibition of the p38 MAPK can reverse the IFNγ-mediated resistance of CLL cells to venetoclax.

However, the position of p38 MAPK within the signaling cascade downstream of IFNγR remained unclear. To address this, we cultured cells derived from five patients with CLL in the presence and absence of 100 ng/mL IFNγ for 1 h. The cells were then harvested and probed for expression of the phosphorylated and total forms of p38 MAPK using immunoblotting. These data show that IFNγ activates the p38 MAPK pathway in these CLL cells (Fig. 5c). The activity of p38 MAPK increased by 40% in cells treated with IFNγ. Thus, this suggested that the prosurvival signals of IFNγ are conveyed from IFNγR in a p38 MAPK-dependent manner. Collectively, this proposes that cytoprotective effects of IFNγ are conveyed through the p38 MAPK pathway and can be overturned with selective inhibitors of p38 MAPK.

### P38 MAPK is overexpressed in venetoclax-resistant CLL cells

Block of the anti-apoptotic activity of Bcl-2 by venetoclax results in cell death. Based on this, we hypothesized that the MEK/ERK1/2 pathway (involved in cell growth and proliferation) and the p38 MAPK and JNK pathways (stress-induced pathways) have roles in the resistance of CLL cells to venetoclax. To shed more light on the molecular mechanisms of venetoclax resistance, the MEC-1 and MEC-1 VER cells were probed for expression of Ras, p-MEK, MEK, p-ERK1/2, ERK1/2, p-p38 MAPK, p38 MAPK, p-JNK, and JNK under basal conditions and following treatment with venetoclax.

**Fig. 6:**
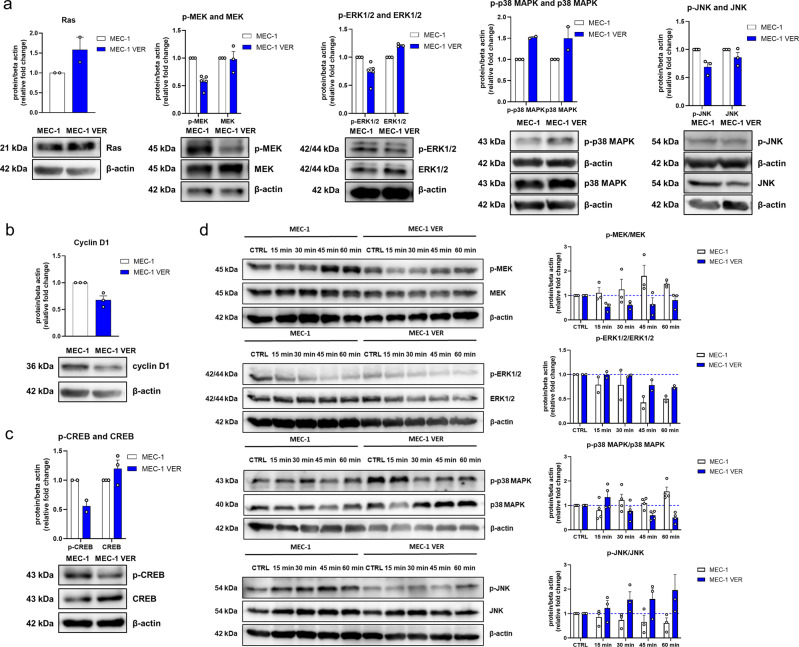
Differential expression of MAPKs and their downstream effectors in MEC-1 and MEC-1 VER cells. **a**–**c** Expression levels of Ras, p-MEK, MEK, p-ERK1/2, ERK 1/2, p-p38 MAPK, p38 MAPK, p-JNK, JNK (**a**), cyclin D1 (**b**), and p-CREB and CREB (**c**) in MEC-1 and MEC-1 VER cells under basal conditions; **d** The expression levels of p-MEK, MEK, p-ERK1/2, ERK1/2, p-p38 MAPK, p38 MAPK, and p-JNK, JNK over time following treatment of MEC-1 and MEC-1 VER cells with 10 µM venetoclax. Data are means ± SEM of ≥ two independent experiments. Representative immunoblots are also shown.

Under basal conditions, compared to the MEC-1 cells, in the MEC-1 VER cells the levels of Ras was increased by 59%, while the levels of p-MEK and p-ERK1/2 were reduced by 43% and 27%, respectively (Fig. 6a). Along with the indicated lower activity of ERK1/2 in the MEC-1 VER cells, they also showed lower levels of p-CREB and thus lower CREB activity, whereby the p-CREB was 56% of that observed for the MEC-1 cells (Fig. 6c). This suggested that MEC-1 VER cells harness another pathway for promotion of cell growth and proliferation, that bypasses the established Ras/MEK/ERK1/2 pathway. In line with this, the levels of the negative regulator of Ras/MEK/ERK1/2 pathway, p38 MAPK were increased by 50% in MEC-1 VER cells (Fig. 6a). This was paralleled by the same proportional increase in the levels of the p-p38 MAPK (by 52%). In line with the increased levels of p38 MAPK and its known activity as a negative regulator of cyclin D1 [43], the levels of cyclin D1 were lower in the MEC1 VER cells (by 32%) (Fig. 6b). In contrast to p38 MAPK, the levels of p-JNK and JNK in MEC-1 VER cells were lower by 32% and 14%, respectively, which is in line with the fact that p38 MAPK is also a negative regulator of JNK [44, 45] (Fig. 6a).

We next investigated how the activities of MAPKs in the MEC-1 and MEC-1 VER cells changed over time (0–60 min) under treatment with 10 µM venetoclax (Fig. 6d). The activity of MEK increased in venetoclax-treated MEC-1 cells and decreased in venetoclax-treated MEC-1 VER cells. However, the activity of ERK1/2 decreased in both the MEC-1 cells and MEC-1 VER cells. Considering the two stress kinases, the activity of p38 MAPK moderately increased in venetoclax-treated MEC-1 cells and was particularly suppressed in the venetoclax-treated MEC-1 VER cells (Fig. 6d). Conversely, the activity of JNK decreased in venetoclax-treated MEC-1 cells, while it increased in venetoclax-treated MEC-1 VER cells. Overall, this provides evidence that these venetoclax-sensitive and venetoclax-resistant cells respond differently to the cytotoxic stress induced by venetoclax.

### Inhibition of p38 MAPK overcomes the resistance of MEC-1 VER cells and patient-derived CLL cells to venetoclax

**Fig. 7:**
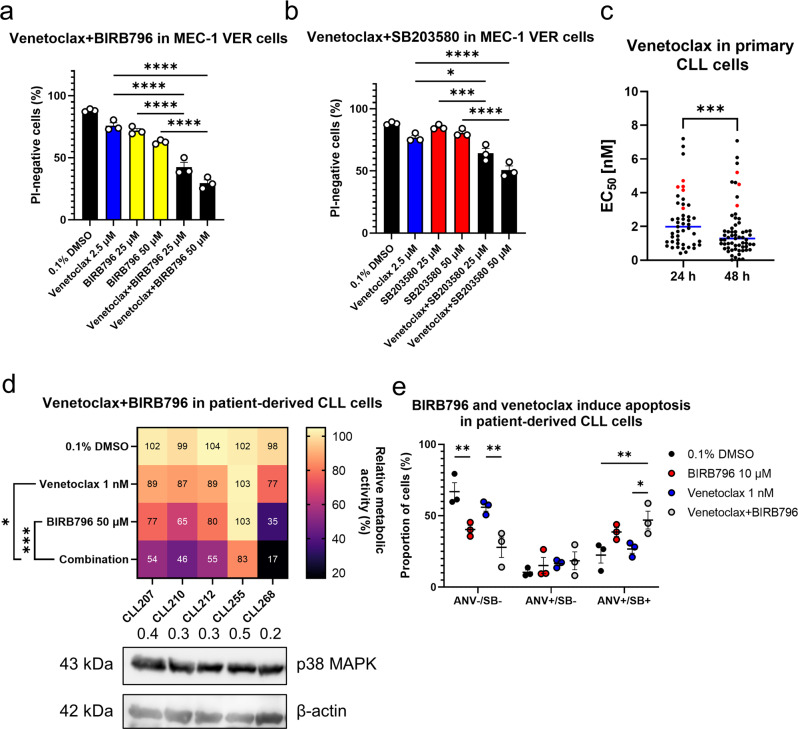
Inhibition of p38 MAPK overcomes the resistance of CLL cells to venetoclax. MEC-1 VER cells (3 × 10^5^ cells/mL) were treated with 0.1% DMSO (vehicle control) or 10 µM venetoclax alone or in combination with 25 and 50 µM BIRB796 (**a**) and 25 and 50 µM SB203580 (**b**) for 48 h. The cells were stained with 5 µM propidium iodide (PI) and the viability of cells (PI-negativity, %) was determined using flow cytometry. Data are means ± SEM of three independent experiments, each carried out in duplicate. One-way ANOVA (post hoc Tukey); *, ***, and **** denote *p* < 0.05, *p* < 0.001, and *p* < 0.0001, respectively. **c** Ex-vivo testing of patient-derived CLL cells to identify those relatively insensitive to venetoclax. CLL cells (1 × 10^6^ cells/mL) from 61 patients with CLL were treated with 0.1–50 nM venetoclax for 24 h and 48 h. The metabolic activities were assessed using the PrestoBlue assay, and then the EC_50_ values were determined using GraphPad Prism 9.2.0. Each symbol corresponds to the individual CLL cell samples. Blue line, median; red symbols, the five CLL cell samples defined as relatively insensitive to venetoclax (EC_50_ ≥ 2-fold the median), as used in (**d**). Paired Student t-test (*n* = 43); *** denotes *p* < 0.001; **d** BIRB796 augments the action of venetoclax in CLL cells that are relatively insensitive to venetoclax. CLL cells (1 × 10^6^ cells/mL) from five CLL patients were treated with 0.1% DMSO (vehicle control) and 1 nM venetoclax, 50 µM BIRB796 and their combination for 24 h. The metabolic activities of cells were determined using the PrestoBlue assay. Data in squares are mean metabolic activities of independent experiments, carried out in duplicate. Rows represent responses of cells from different CLL patients to a specific treatment, while columns represent responses of cells from a specific CLL patient to different treatments. One-way ANOVA (post hoc Dunnett); * and *** denote *p* < 0.05 and *p* < 0.001, respectively. Immunoblot of CLL samples probed for the expression of p38 MAPK and β-actin is shown. **e** Detection of apoptosis after 24-hour treatment of primary cells (1 × 10^6^ cells/mL) with 10 µM BIRB796, 1 nM venetoclax, and their combination. Data are means ± SEM of three independent experiments. Two-way ANOVA (post hoc Tukey); * and ** denote *p* < 0.05 and *p* < 0.01, respectively.

Given that p38 MAPK was overexpressed in the MEC-1 VER cells, we reasoned that addition of a p38 MAPK inhibitor can overcome the resistance of these MEC-1 VER cells to venetoclax. To test this, MEC-1 VER cells were treated with 2.5 µM venetoclax alone and in combination with 25 and 50 µM BIRB796 or 25 and 50 µM SB203580 for 24 h. The viabilities of these cells were then determined using propidium iodide and flow cytometry (Fig. 7).

The viability of the MEC-1 VER cells treated with 2.5 µM venetoclax and 25 µM BIRB796 decreased from 88% (vehicle control) to 76% and 72%, respectively, while their combination decreased the viability further, to 43% (Fig. 7a). Similarly, the viability of the MEC-1 VER cells treated with 2.5 µM venetoclax and 50 µM SB203580 decreased from 88% (vehicle control) to 77% and 81%, respectively, while their combination decreased this further, to 51% (Fig. 7b). Collectively, this demonstrated that inhibition of p38 MAPK can restore the sensitivity of MEC-1 VER cells to venetoclax, thus suggesting that this represents a new strategy for treating patients with venetoclax-resistant CLL.

To further support this, we initially screened a set of patient-derived CLL cells (*n* = 61) from the CLL biobank (*n* = 362) to identify those that responded poorly to venetoclax (Fig. 7c). These CLL cells were treated with 0.1–50 nM venetoclax for 24 h and 48 h, with determination of their metabolic activities using the PrestoBlue assay. The EC_50_ values for venetoclax were then calculated using the GraphPad Prism 9.2.0, which showed means of 2.3 nM (*n* = 47) and 1.7 nM (*n* = 61), respectively, whereby venetoclax demonstrated greater cytotoxicity after 48 h (*n* = 43; *p* = 0.0002) (Fig. 7c). The insensitive cells were then defined empirically; as those were the EC_50_ of venetoclax for the individual patient sample was ≥2-fold the median EC_50_. The incidence of insensitivity was 15% (7/47 samples for 24-h and 9/61 samples for 48-h). This thus suggested that such ex-vivo screening can be used to predict the likelihood of relapses of patients with CLL treated with venetoclax.

We then took CLL cells that showed EC_50_ values for venetoclax ≥2-fold the median (Fig. 7c, red dots) and treated these with 1 nM venetoclax, 50 µM BIRB796, and their combination for 24 h (Fig. 7d). While responses of these CLL cells (*n* = 5) to venetoclax were poor (mean response, 89%), the responses to BIRB796 were more variable (mean response, 72%) and predicted the level of cytotoxicity in the combination treatments (mean response, 51%). We then probed these CLL samples for the expression of p38 MAPK using immunoblotting and found that samples with lower levels of p38 MAPK responded better to venetoclax, BIRB796, and their combination. Conversely, samples with higher levels of p38 MAPK responded poorly to treatments. To gain a mechanistic insight into the synergistic cytotoxicity of of BIRB796 and venetoclax in CLL, we treated cells derived from three patients with CLL with 0.1% DMSO, 10 µM BIRB796, 1 nM venetoclax, and their combination for 24 h (Fig. 7e) and then used the SYTOX Blue/annexin V assay to assess the level of apoptosis. The viabilities of cells (ANV-/SB-) treated with 0.1% DMSO, BIRB796, venetoclax, and their combination were 67%, 40%, 56%, and 28%. The proportion of late-apoptotic (ANV + /SB + ) cells increased accordingly from 22% for vehicle control to 39%, 27%, and 47% for cells treated with BIRB796, venetoclax, and their combination, respectively. Thus, these data show that the pharmacological block of p38 MAPK augments the action of venetoclax via triggering of apoptosis and can abrogate the venetoclax insensitivity of primary CLL cells, which suggests that such approach can be applied in vivo.

## Discussion

We have investigated here the molecular mechanisms that drive venetoclax resistance in patients with CLL, with the focus being to provide new strategies to successfully combat it. Using IFNγ, PMA/ionomycin, and sCD40L we mimicked the clonal stimulation that occurs in the tumor microenvironment of CLL cells. IFNγ is present in the blood of CLL patients and is also found in the tumor microenvironment [9–11], where it can activate CLL cells in both paracrine and autocrine manners [11]. We thus initially demonstrated that IFNγ can activate CLL cells and rescue them from spontaneous and venetoclax-induced apoptosis. This is in line with previously published studies, that have reported the stimulating and anti-cell death properties of IFNγ on CLL cells [11–14].

PMA mimicks diacylglycerol and is therefore a direct activator of protein kinase C. PMA can induce activation and differentiation of CLL cells into IgM-secreting plasma cells, as reviewed by [46], and it is commonly used in conjunction with the calcium ionophore ionomycin to induce activation and proliferation of normal B cells [47, 48]. Mimicking BCR activation in CLL cells with PMA/ionomycin provided significant protection against venetoclax cytotoxicity and induced nuclear translocation of NFκB. As PMA/ionomycin activated the PKC/NFκB pathway and abrogated venetoclax cytotoxicity, we reasoned that stimuli that converge on the NFκB pathway are important for survival and resistance of CLL cells to venetoclax. This is supported by the previously established roles of BCR and NFκB signaling in the survival of CLL cells and strengthens the implication of BCR and NFκB in fostering resistance to targeted therapies [18, 27, 28].

CD40L-mediated activation of NFκB has been shown to foster resistance to venetoclax [29, 30]. However, while the ligation of CD40 triggered translocation of NFκB to the nucleus in primary CLL cells, it offered only minimal protection against venetoclax cytotoxicity, thus proposing that some other pathways aside from the NFκB are responsible for venetoclax resistance. One such pathway might be the p38 MAPK pathway, which is required for CD40-induced NFκB activation in B lymphocytes [32].

Given that the entire population of IFNγ-primed cells was rescued from venetoclax-induced apoptosis, we reasoned that IFNγ-mediated signaling is an even more important mechanism of resistance of CLL cells to venetoclax than those already shown to be due to CD40 and BCR signaling [18, 31]. These effects of IFNγ are likely to be transduced via the JAK/STAT pathway [14, 20], which is implicated in resistance to contemporary targeted drugs used for the treatment of patients with CLL [16]. In particular, IFNγ can abolish venetoclax cytotoxicity via the JAK-Src/STAT3/Mcl-1 pathway, whereby this leads to upregulation of Mcl-1 in CLL cells [14], which is a well know resistance mechanism of CLL cells to venetoclax [17, 18]. However, IFNγ has other cellular effects that are independent of the JAK/STAT pathway [20]. We investigated the known ability of IFNγ to induce the formation of the immunoproteasome [21, 22]. We demonstrated that following the treatment of MEC-1 cells with IFNγ, the immunoproteasome activity increased in a time-dependent manner. IFNγ also activated immunoproteasome in patient-derived CLL cells. The immunoproteasome was successfully targeted with the selective inhibitor ONX-0914, which suppressed the activity of immunoproteasome and synergized with venetoclax against primary CLL cells, and successfully tackled IFNγ-mediated resistance of patient-derived cells to venetoclax. These findings highlight the potential of immunoproteasome as a target in venetoclax-resistant CLL.

Aside from the activation of the immunoproteasome, we also found that IFNγ activates the p38 MAPK pathway. Pharmacological inhibition of p38 MAPK abolished the stimulating and resistance-promoting properties of IFNγ in CLL cells, thus restoring the cytotoxicity of venetoclax. To provide more comprehensive insight into the role of p38 MAPK in driving resistance to venetoclax, we established venetoclax-resistant CLL cells: MEC-1 VER cells. We then investigated the levels of anti-apoptotic proteins in these cells, which are implicated in the resistance of CLL cells to venetoclax [17, 37]. Here, the Bcl-2 and Bcl-xL levels did not differ between MEC-1 and MEC-1 VER cells, while Mcl-1, Bax, and Bak were down-regulated in venetoclax-resistant cells. In contrast, p-Bcl-2 (Ser70) and Bid were upregulated. This indicated that resistance of the MEC-1 VER cells to venetoclax was not Mcl-1–mediated or Bcl-xL mediated, but was rather due to a decrease in proapoptotic effectors and higher phosphorylation of Bcl-2, which enhances its anti-apoptotic activity [49, 50]. We then investigated the expression and activity of four MAPKs under basal and venetoclax-treated conditions. Under basal conditions, Ras and p38 MAPK were up-regulated compared to normal cells, while the activities of the MEK/ERK1/2 pathway and the JNK pathway were decreased. It has been shown that p38 MAPK inhibits the JNK pathway through induction of the phosphatase DUSP1 [44], which along with DUSP6, was recently reported to control the activity of ERK in CLL cells. Inhibition of these phosphatases led to hyperphosphorylation of ERK and induction of cell death that specifically occurred in CLL cells when compared to normal or other types of B-cell lymphoma cells [51]. In line with this, the MEC-1 VER cells overexpressing p38 MAPK showed decreased expression of JNK and decreased activity of ERK, potentially through the same DUSP1-dependent mechanism as reported previously [44]. We thus propose that p38 MAPK is a driver of venetoclax resistance in these MEC-1 VER cells, and secondly that JNK kinase in these MEC-1 VER cells is down-regulated, to thus circumvent the cell death that JNK kinase has been reported to trigger under stress conditions [52].

Huelsemann et al. reported that activation of p38 MAPK reduces the expression of Mcl-1 [37], which supports our findings here where there was up-regulation of p38 MAPK with down-regulation of Mcl-1 in the venetoclax-resistant MEC-1 VER cells. Moreover, Chiou et al. recently demonstrated that Mcl-1 is downregulated via p38 MAPK mediated dephosphorylation of CREB [53], which is in line with our findings, whereby p38 MAPK is upregulated and p-CREB and Mcl-1 are decreased. We thus postulated that p38 MAPK is responsible for resistance to venetoclax. Indeed, pharmacological inhibition of p38 MAPK with both BIRB796 and SB203580 was able to overcome the resistance of MEC-1 VER cells to venetoclax and also demonstrated potent synergistic cytotoxicity against venetoclax-insensitive primary CLL cells. This supports the findings that p38 MAPK is important for maintenance of CLL cells [35, 36], but contradicts the proapoptotic nature of p38 MAPK in CLL [37–39]. These differences can in part be explained by the participation of p38 MAPK in numerous cellular processes, as it is also the negative regulator of other MAPKs, and in part by the higher concentrations of p38 MAPK inhibitors used in the present study.

## Supplementary information

Supplemental material

Reproducibility checklist

## Data Availability

The data presented in this study are available upon reasonable request from the corresponding author. The data are not publicly available due to privacy and ethical restrictions.
