# Supplementary material for: Inhibition of p38 MAPK or immunoproteasome overcomes resistance of chronic lymphocytic leukemia cells to Bcl-2 antagonist venetoclax
Source: Cell Death Dis. 2022 Oct 8;13(10):860. doi: 10.1038/s41419-022-05287-6 (PMC9547871; doi:10.1038/s41419-022-05287-6)

Full and uncropped immunoblots for Figure 3c

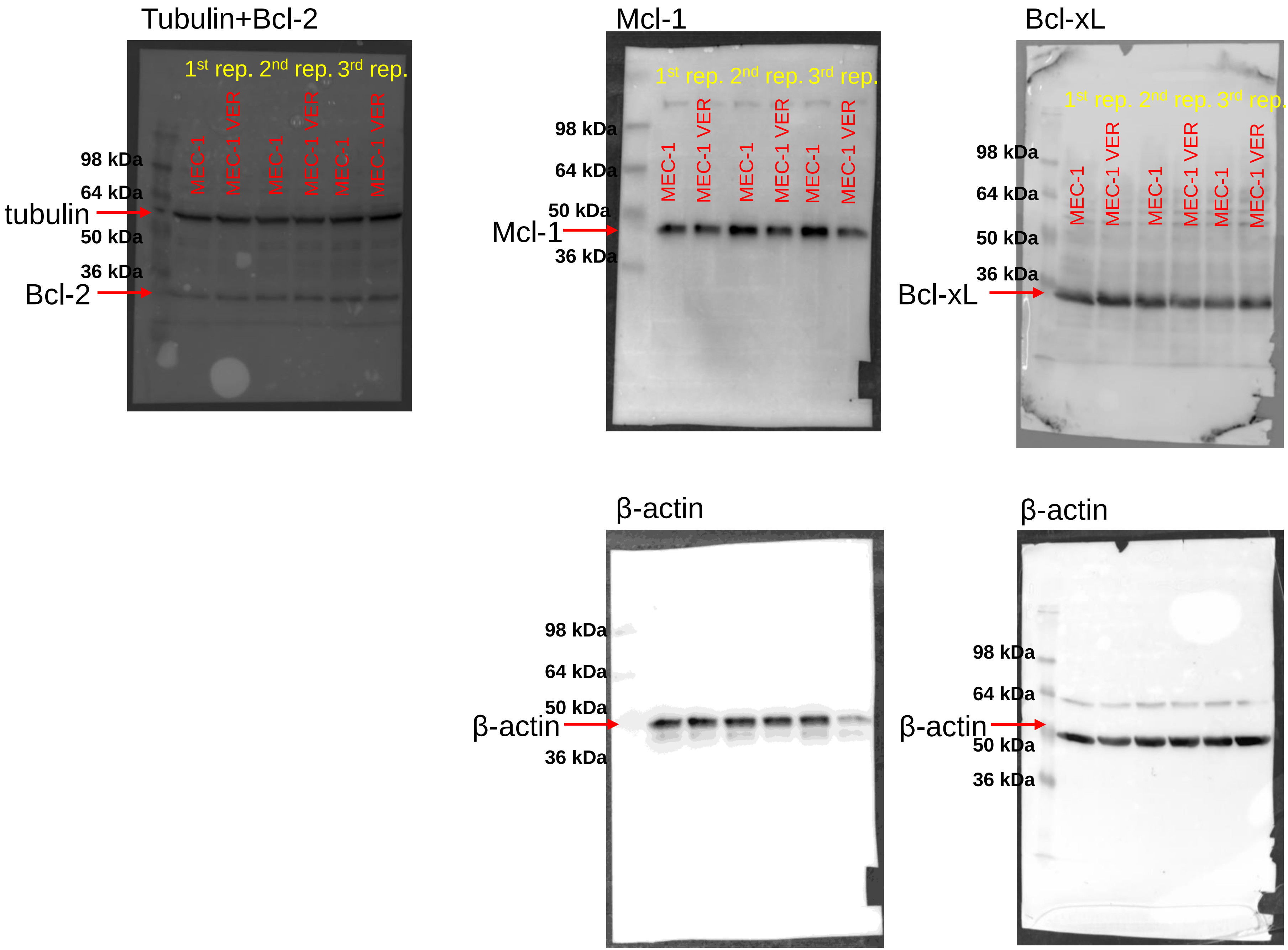

Full and uncropped immunoblots for Figure 3c

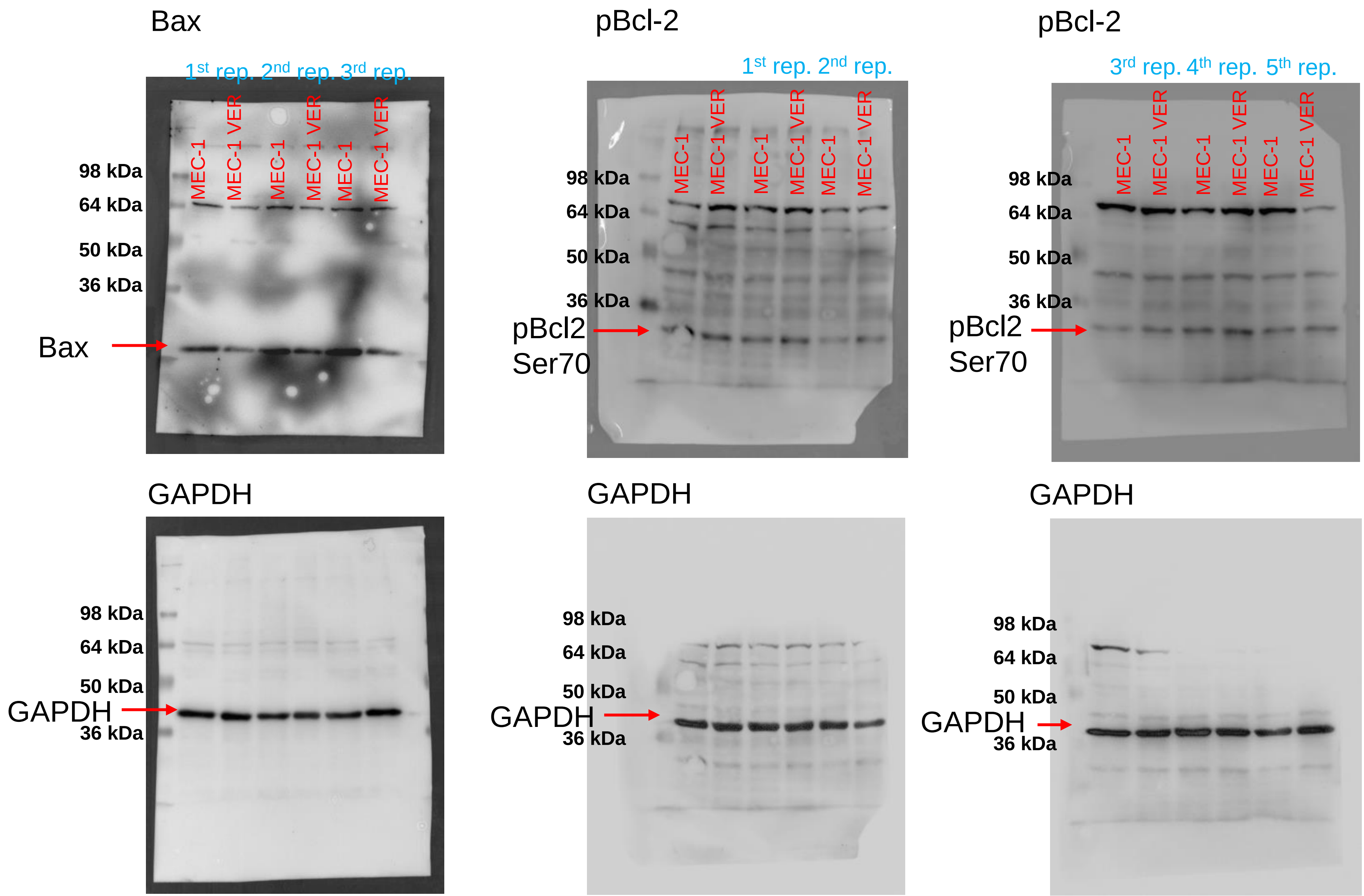

Full and uncropped immunoblots for Figure 3c

Bid

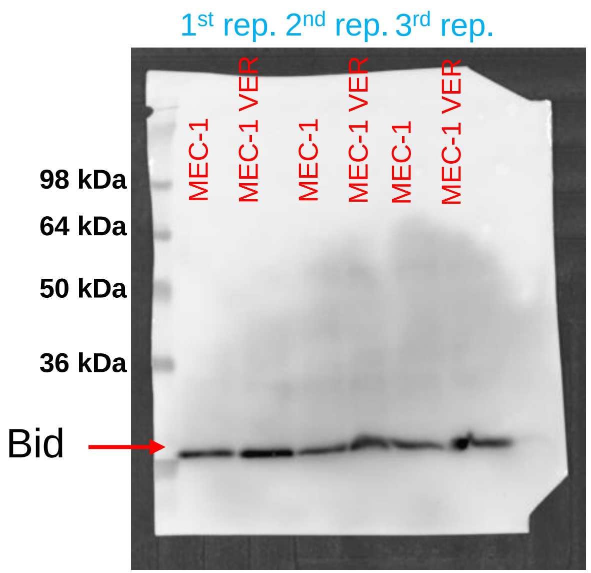

PUMA

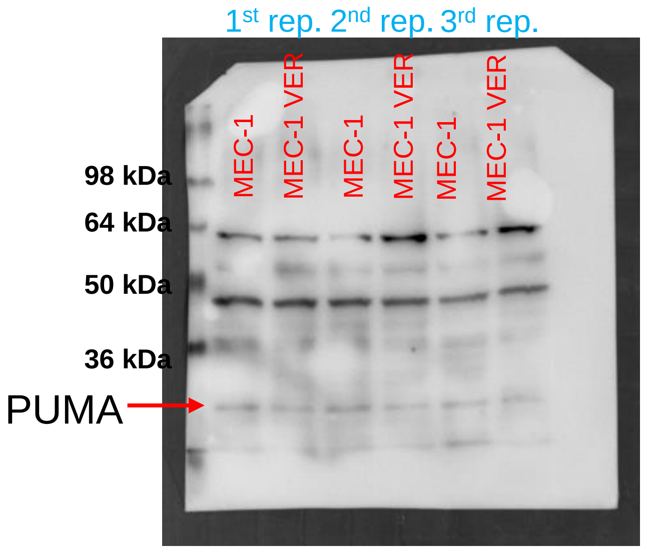

Bak

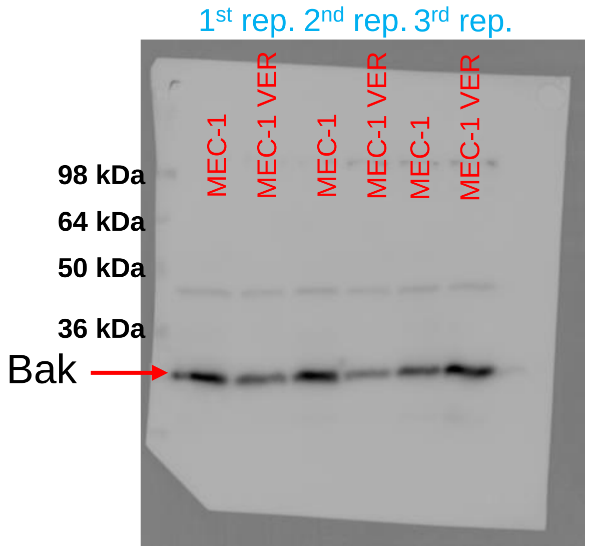

GAPDH

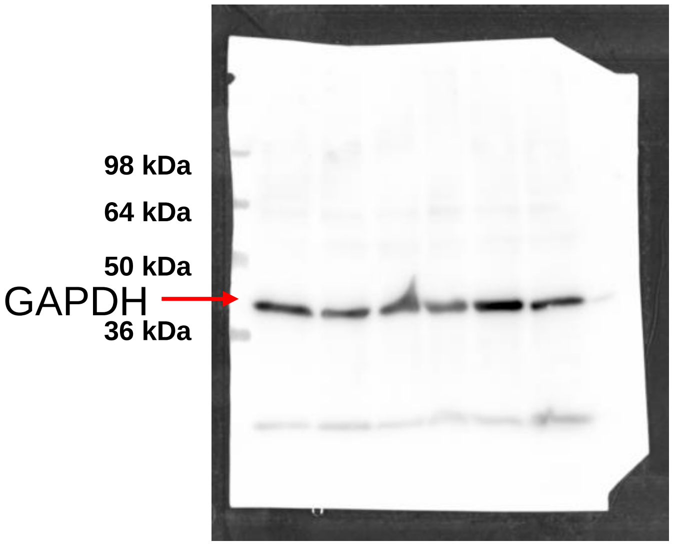

GAPDH

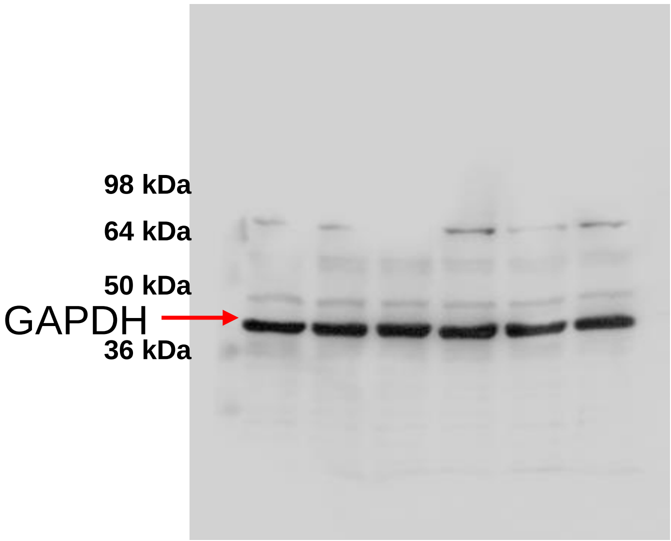

GAPDH

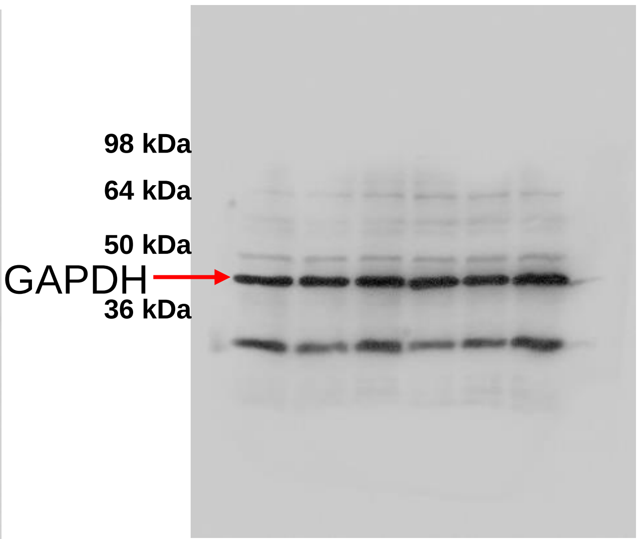

Full and uncropped immunoblots for Figure 5c

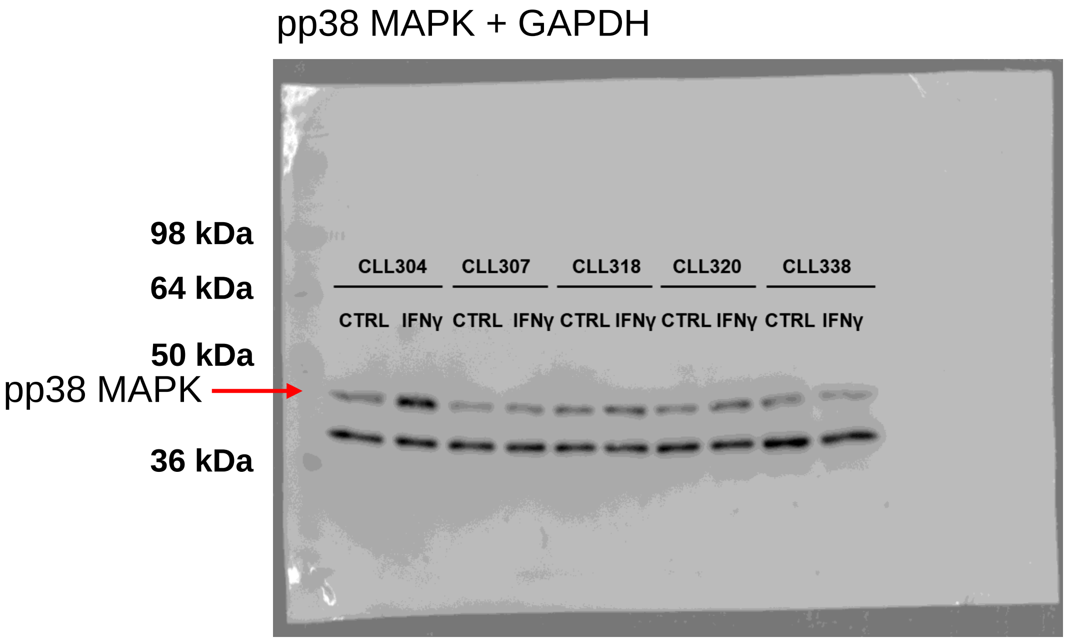

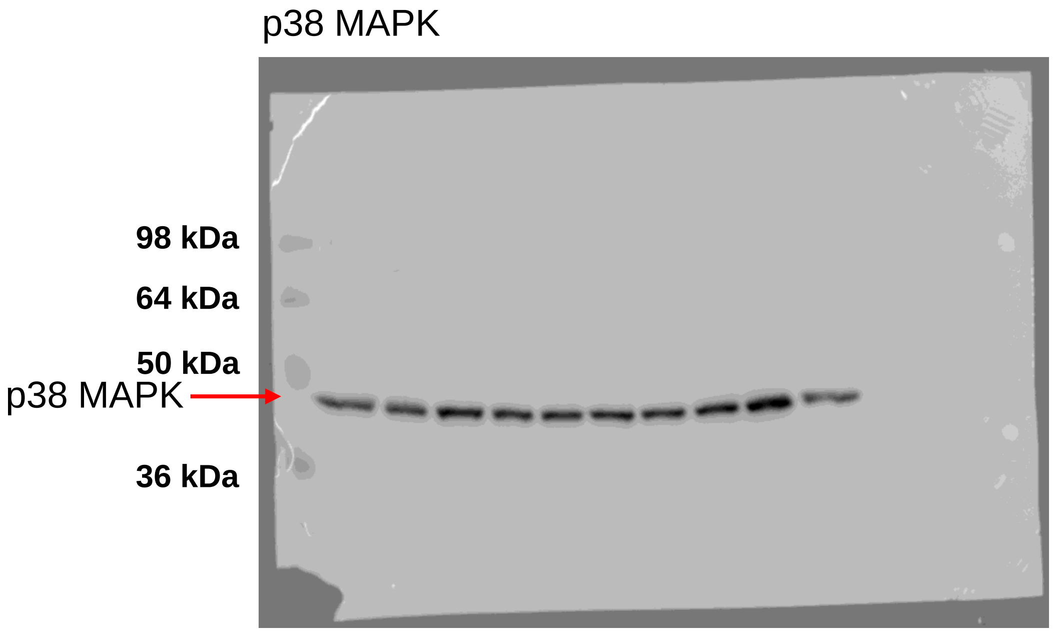

Full and uncropped immunoblots for Figure 6a

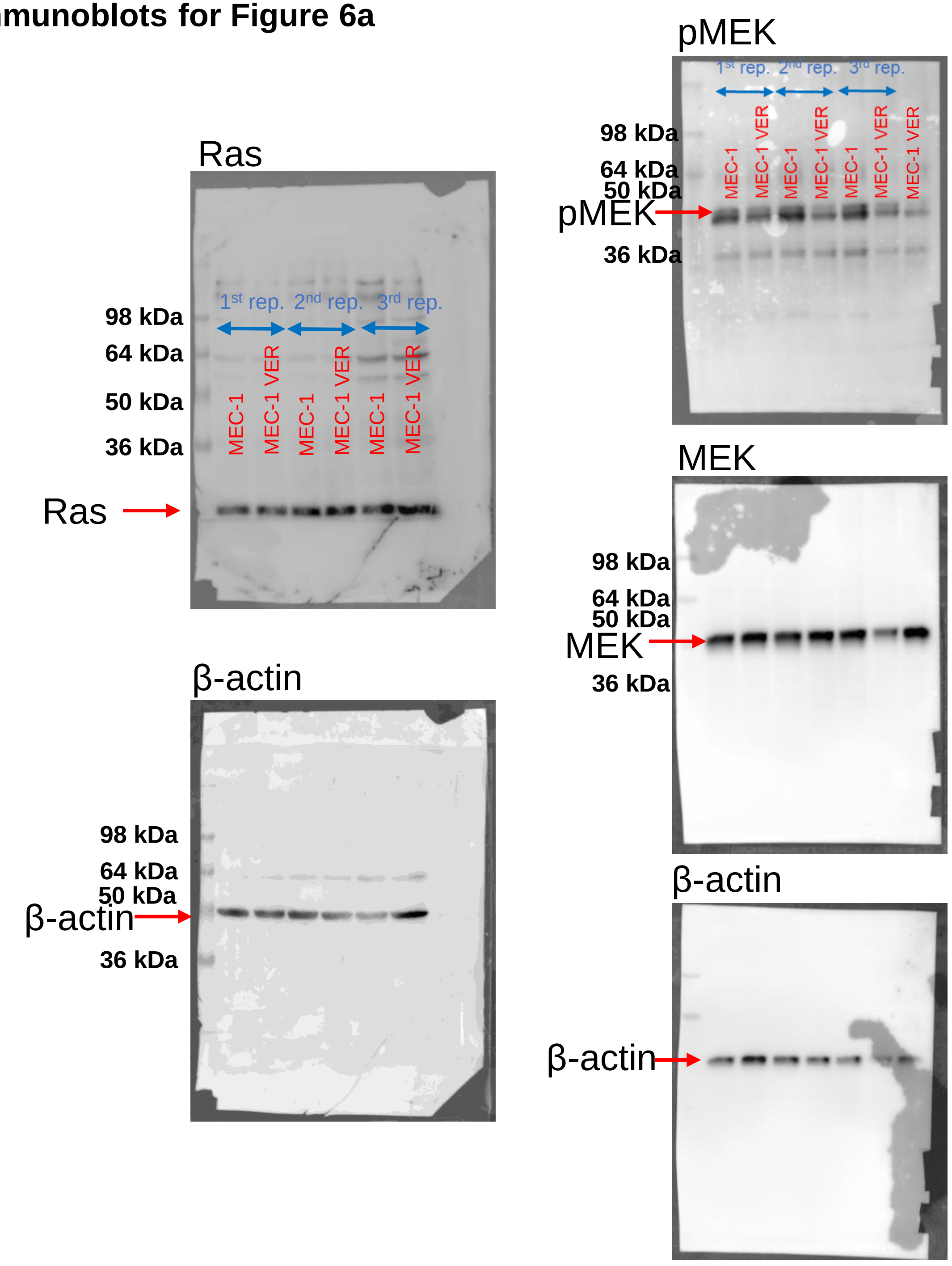

Full and uncropped immunoblots for Figure 6a

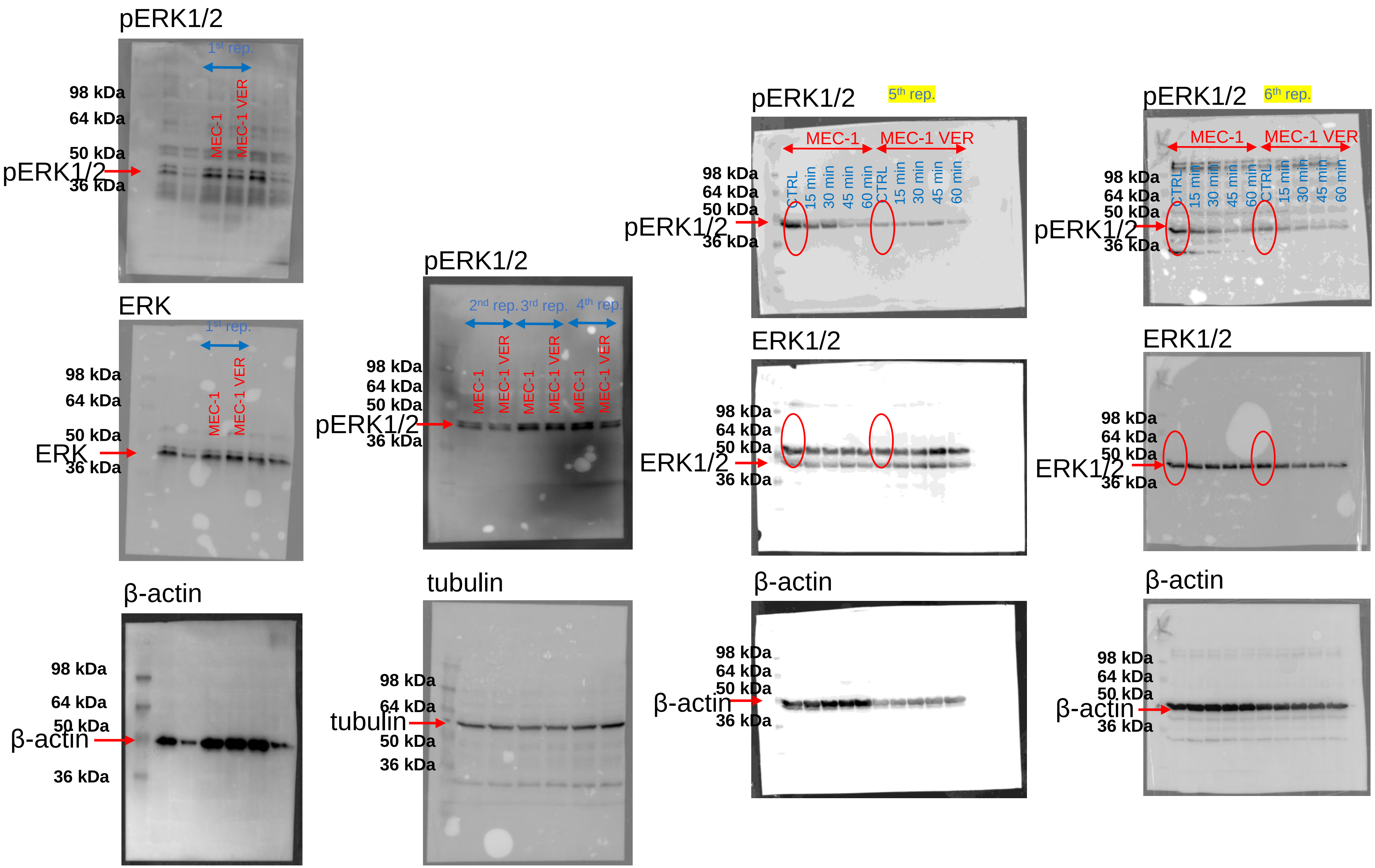

Full and uncropped immunoblots for Figure 6a

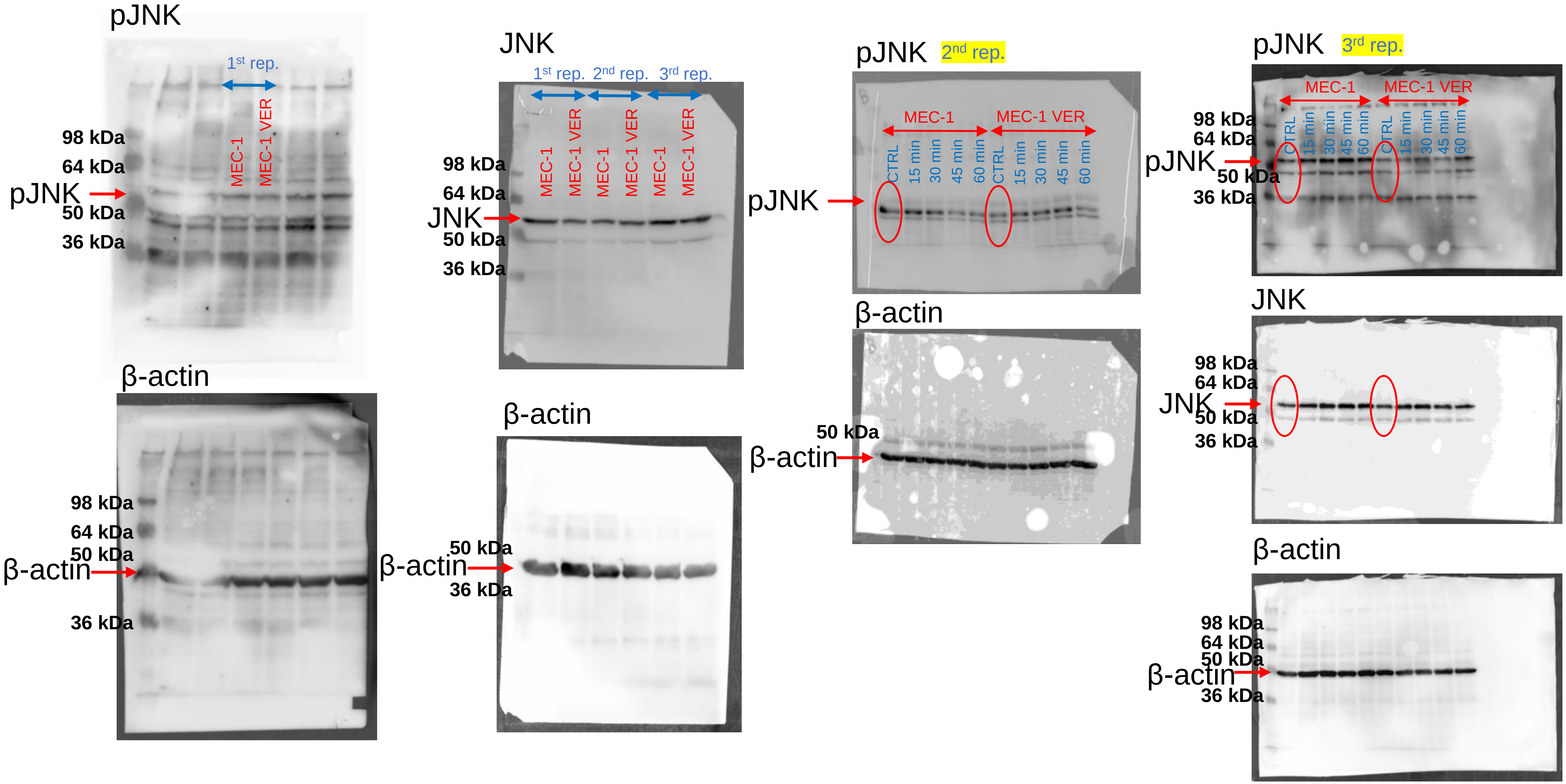

Full and uncropped immunoblots for Figure 6a-c

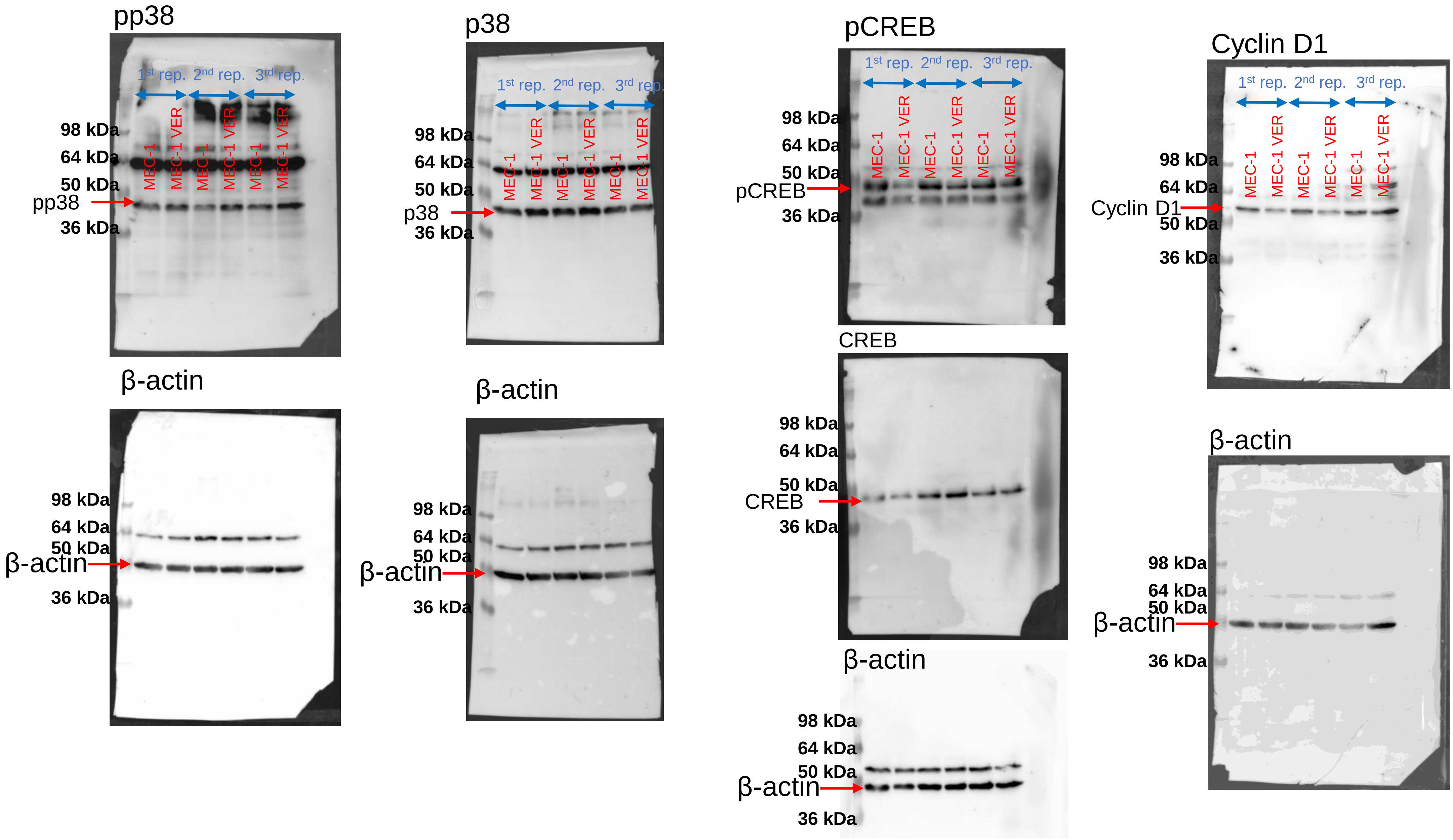

Full and uncropped immunoblots for Figure 6d

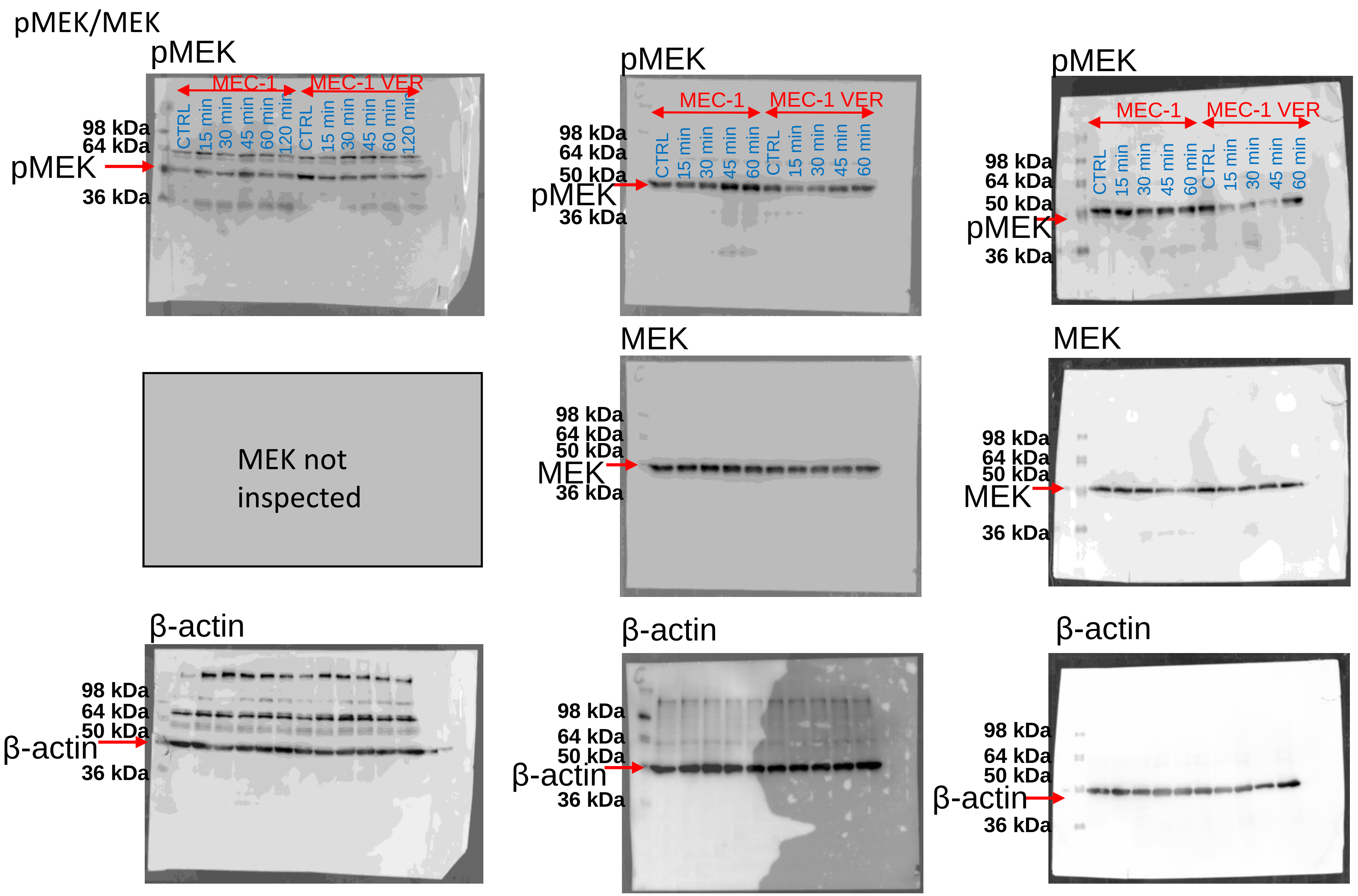

Full and uncropped immunoblots for Figure 6d

pERK/ERK

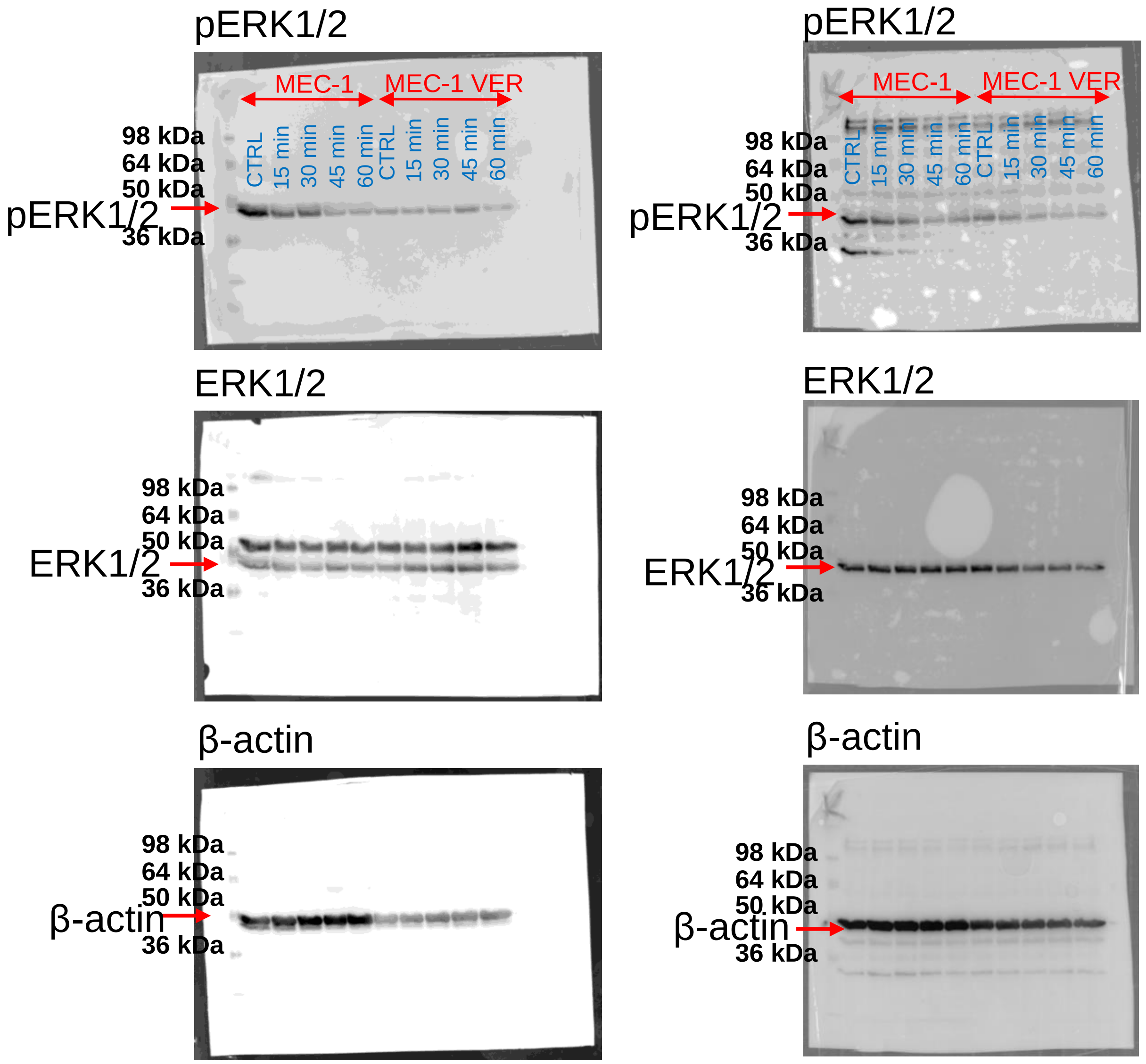

Full and uncropped immunoblots for Figure 6d

pp38 MAPK/p38 MAPK

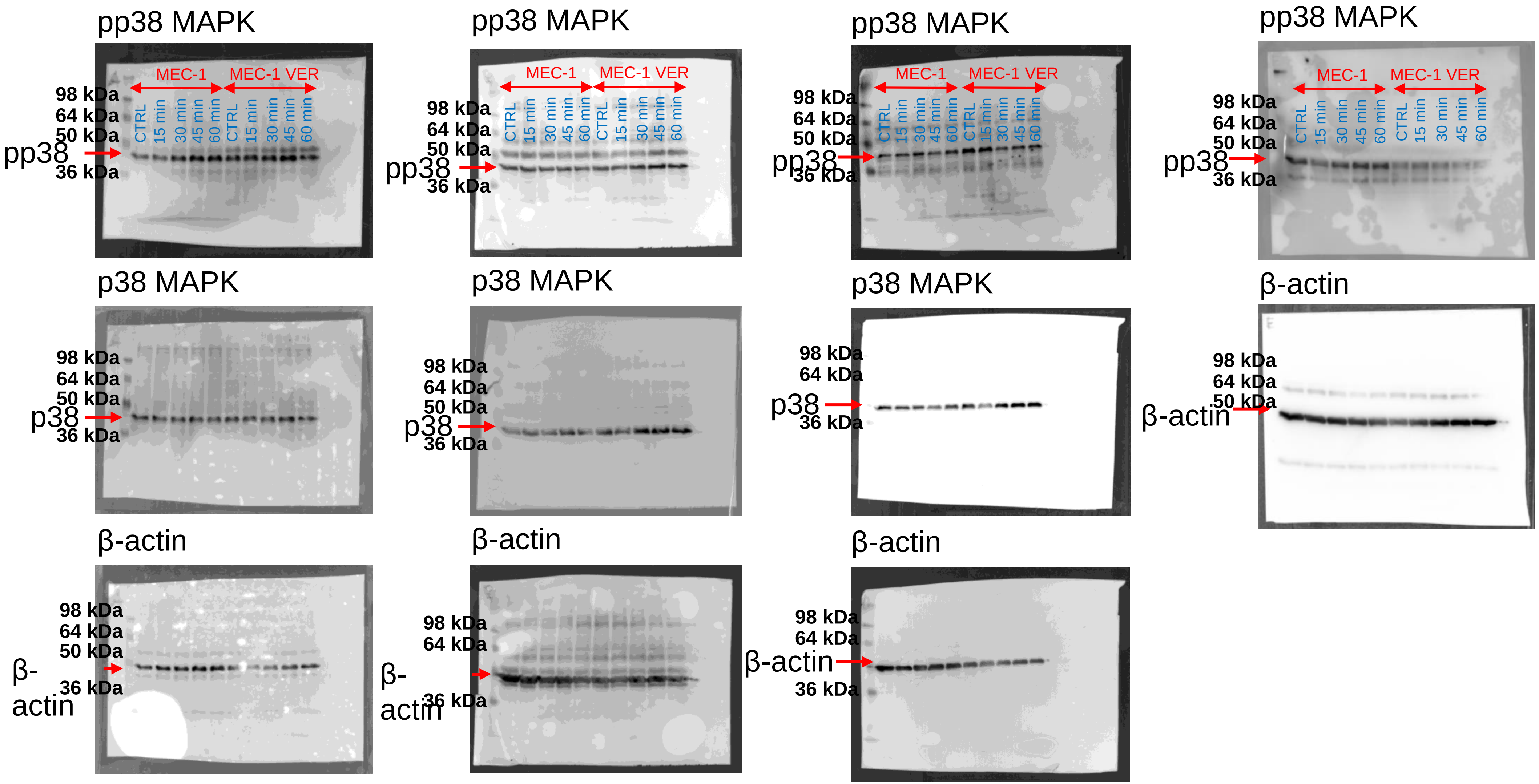

Full and uncropped immunoblots for Figure 6d

pJNK/JNK

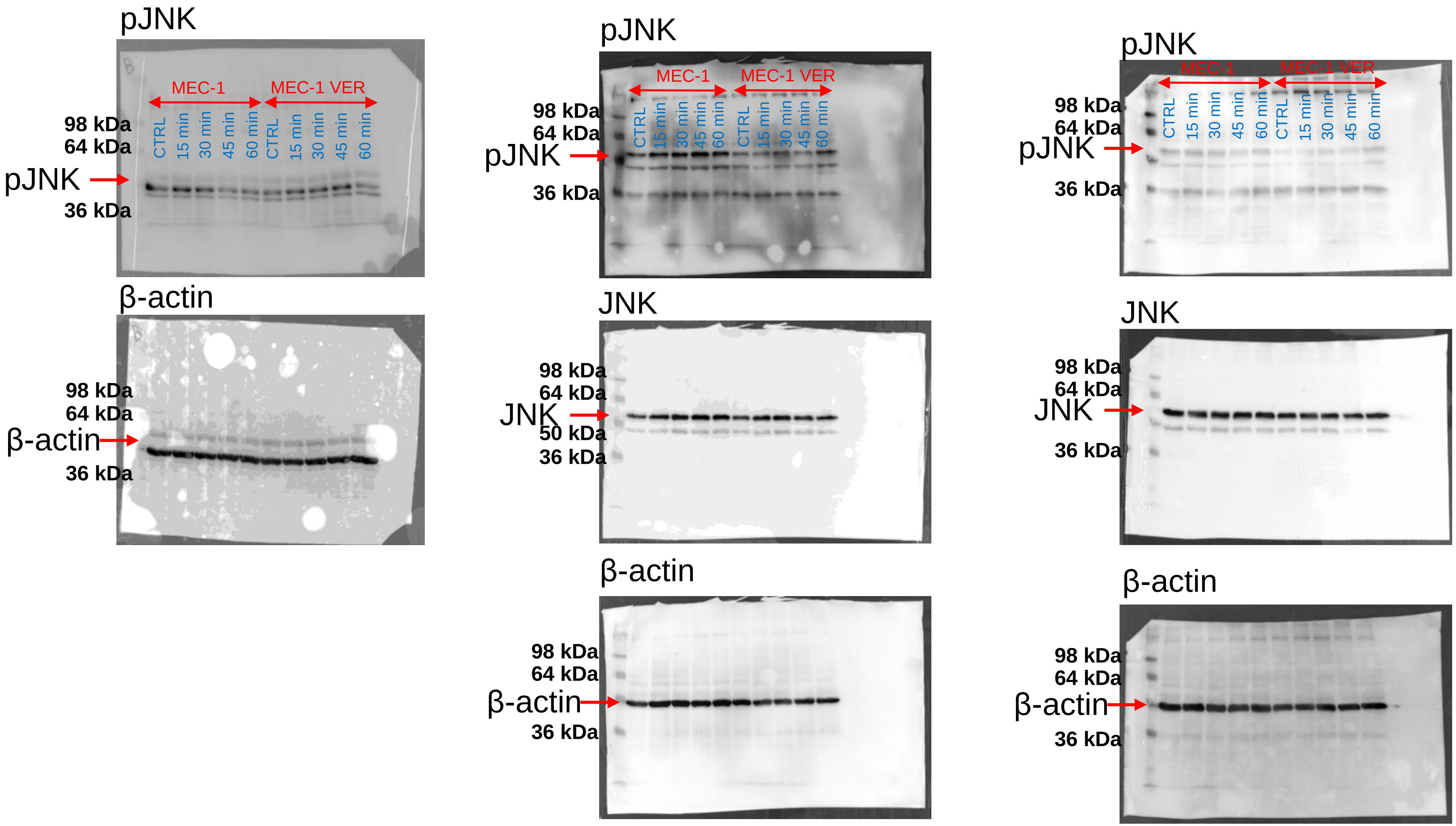

Full and uncropped immunoblots for Figure 7d

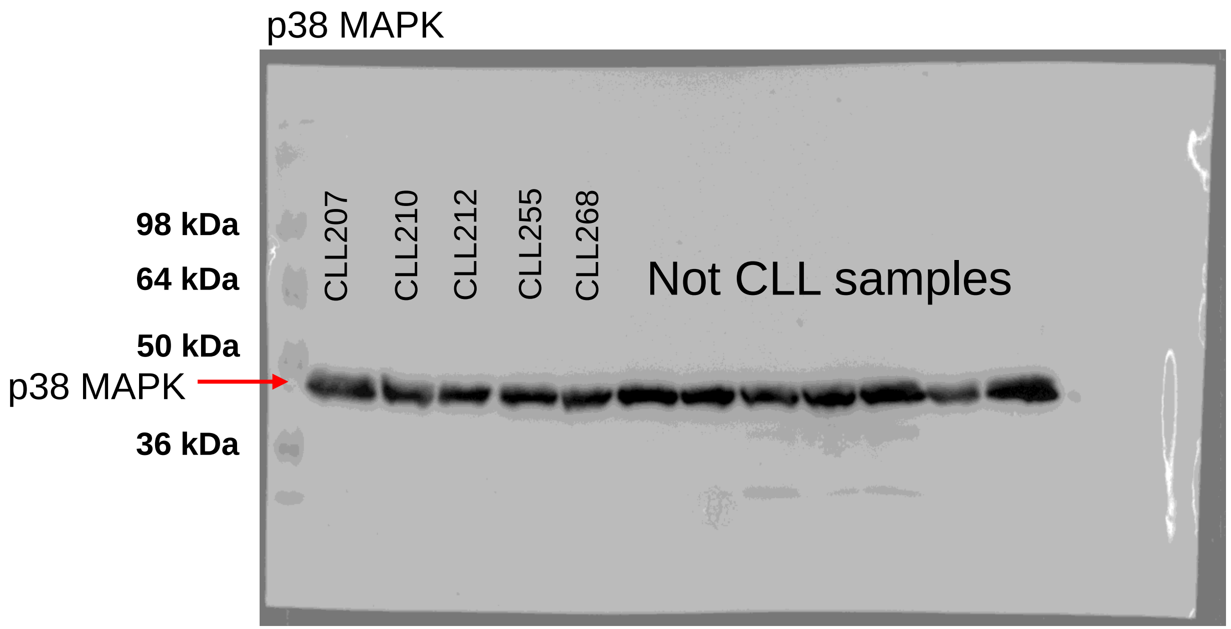

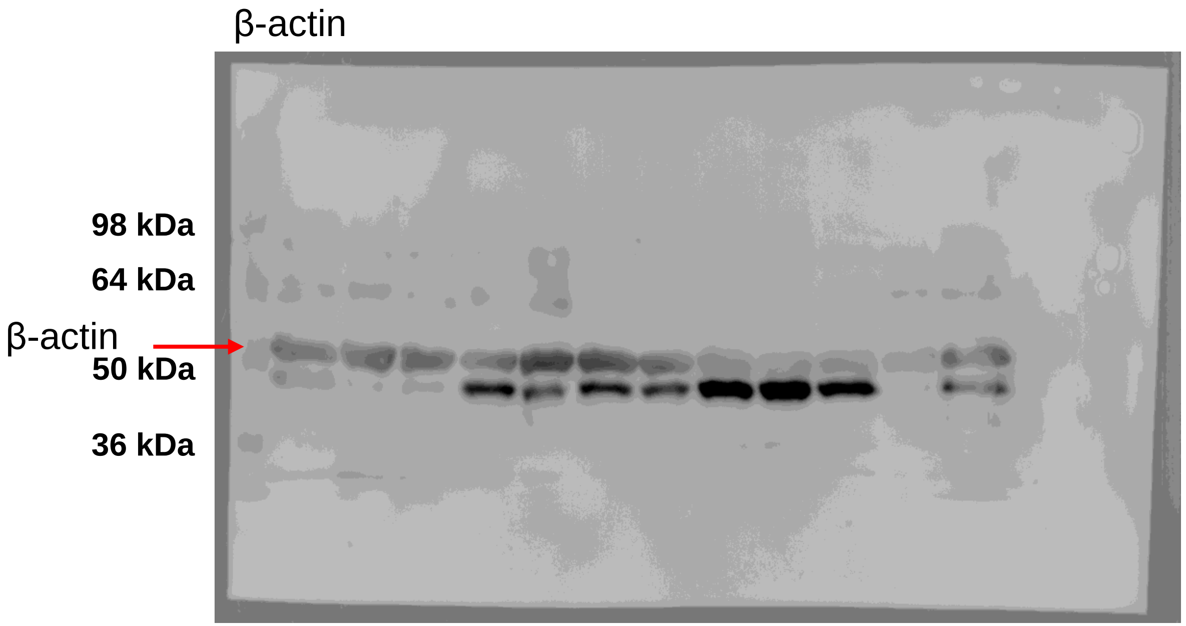

Supplement: Supplementary file 1 — Supplemental material [file 41419_2022_5287_MOESM1_ESM.pdf]
